# Enhancing Gluten‐Free Bread With Whole Flours From Forage Palm, Buckwheat, and Teff: Physicochemical Composition, Technological Properties, and Sensory Evaluation

**DOI:** 10.1111/1750-3841.70812

**Published:** 2026-01-05

**Authors:** Mateus Alves Araújo, Nathalia de Andrade Neves, Irene Andressa, Tatiana Nunes Amaral, Marcio Schmiele

**Affiliations:** ^1^ Institute of Science and Technology Federal University of Jequitinhonha and Mucuri Valleys (UFVJM) Diamantina Brazil; ^2^ Department of Food Technology Federal University of Viçosa (UFV) Viçosa Brazil

**Keywords:** bioactive compounds, innovation, new flours, pseudocereals, sustainability, texture

## Abstract

The increasing demand for gluten‐free foods has driven the formulation of baked products using alternative raw materials to replace wheat. This study aimed to characterize the whole flours of forage palm, teff, and buckwheat, as well as evaluate their performance in gluten‐free breads. Proximate analyses of the flours were conducted, in addition to the characterization of the batters and the breads developed. The results indicated that buckwheat flour exhibited the highest protein content (11.58%), forage palm flour stood out for its elevated fiber and ash content (17.92%), and teff flour presented the highest total carbohydrate content (77.06%). Based on the obtained data, optimized bread formulations (with and without teff flours) were developed and subjected to proximate and sensory analyses. Both formulations exhibited similar compositions regarding moisture, proteins, and lipids, although they significantly differed in dietary fiber content, which was higher in the formulation without teff (11.44%) compared to the one with teff (8.56%). Sensory evaluation revealed greater acceptance of the formulation containing teff, attributed to its softer texture and more pronounced flavor. The color of the bread varied subtly due to the presence of forage palm whole flour, without compromising appearance and aroma attributes, which were similarly well rated in both formulations.

## 1 Introduction

The growing interest in gluten‐free diets has been stimulated not only by the increasing demand for novel food products, particularly within bakery goods (Spizzirri et al. 2022), but also by consumers who perceive these products as part of a healthier lifestyle. The main disorders associated with gluten consumption, including celiac disease, gluten ataxia, wheat allergy, and non‐celiac gluten sensitivity, are estimated to affect approximately 3% of the global population (Wang et al. 2020). For individuals affected by these conditions, the only effective treatment has been recognized as strict adherence to a gluten‐free diet, which promotes clinical recovery, achieves microvilli restoration, and improves gastrointestinal function (Asri et al. 2021; Šmídová and Rysová 2022; Peñalver et al. 2023). In addition, the development of gluten‐free bakery products provides an opportunity to enhance the nutritional profile by incorporating fibers, bioactive compounds, proteins, and essential minerals such as iron, thus meeting both health‐related needs and consumer expectations for more balanced food options. The substitution of wheat in food formulations presents significant technological, nutritional, and sensory challenges. Commonly used alternatives include rice, corn, sorghum, and quinoa flours, which, in addition to being free of gluten‐forming proteins, offer distinct nutritional profiles characterized by higher contents of dietary fiber, protein, and micronutrients (Khairuddin and Lasekan 2021). To compensate for the absence of gluten, an essential protein complex responsible for forming the three‐dimensional matrix in baked products, ingredients referred to as gluten network mimetics are frequently incorporated.

In gluten‐free baking, product quality is closely linked to fermentation performance, structure formation, and sensory characteristics, all of which determine the consumer's overall experience. Batter rise during fermentation reflects the development of the internal structure and gas retention capacity, influencing the bread's lightness and texture. Texture, including softness and firmness, as well as appearance and internal structural uniformity, affects both sensory acceptance and product shelf life (de Oliveira Teotônio et al. 2021). Among the structuring agents commonly used in gluten‐free formulations, hydrocolloids such as xanthan gum, guar gum, agar, carrageenan, hydroxypropyl methylcellulose, and carboxymethylcellulose (Culetu et al. 2021) are applied to replicate gluten functions, improving viscosity, gas retention, and batter stability. Plant‐derived mucilages have also shown potential as natural alternatives, including psyllium, flaxseed (Elsorady et al. 2024), chia (Pontes et al. 2020), and cactus (Salem et al. 2024), enhancing technological and structural characteristics of gluten‐free batters. Understanding these parameters is essential to address the challenges of gluten‐free baking and to enable innovative formulations that incorporate new ingredients, improving both functionality and sensory acceptance of the products.

Among alternative and innovative ingredients, buckwheat (*Fagopyrum esculentum* and *Fagopyrum tataricum*) has been emphasized due to its substantial content of starch, proteins, dietary fibers, lipids, vitamins, minerals, and phenolic compounds such as rutin and quercetin, which possess notable antioxidant and anti‐inflammatory properties (Huda et al. 2021; Kreft et al. 2020; Sofi et al. 2023; Zhu 2021; Zargar et al. 2024). Teff (*Eragrostis tef*), a traditional pseudocereal native to Ethiopia, has been recognized for its balanced nutritional profile and is characterized by elevated levels of iron, calcium, and zinc, as well as the presence of bioactive compounds with functional potential (Dame 2020; Gebru et al. 2020; Habte et al. 2022). Forage palm (*Opuntia ficus‐indica*), cultivated in arid environments such as the Brazilian semiarid region, has been identified as a relevant source of dietary fiber, vitamin C, betalains, and other antioxidant compounds, contributing to glycemic and lipid regulation, in addition to supporting digestive system health (Xavier et al. 2021).

In addition to its nutritional and functional profile, forage palm (*O. ficus‐indica*) has been distinguished by its high mucilage content, primarily composed of hydrophilic polysaccharides. These compounds exhibit a pronounced water‐holding capacity, which may play a pivotal role in enhancing the technological quality of gluten‐free products. This property facilitates batter hydration during processing, contributing to the formation of more homogeneous internal structures and resulting in bread crumbs with improved softness, cohesiveness, and uniformity, attributes often compromised in the absence of the gluten protein network. Moreover, the inclusion of forage palm in gluten‐free formulations represents an innovative approach that combines technological improvements with nutritional enhancements, reinforcing its potential as a promising ingredient for healthier baked products (de Andrade Alves et al. 2021; da Silva et al. 2024).

Despite recent advancements, gluten‐free products available on the market still present limitations in terms of texture, flavor, and nutritional value, mainly due to the absence of gluten, whose functional properties are essential for batter cohesiveness, elasticity, and structural integrity. To overcome these challenges, the use of technologically functional ingredients has been recognized as a key strategy, as they can improve batter behavior and the structural and sensory quality of the final product. Among these, enzymatic treatments such as transglutaminase (TGase) have attracted particular attention, as they promote protein cross‐linking and strengthen the batter network, enhancing both technological performance and nutritional value. The integration of functional ingredients with TGase thus enables the development of gluten‐free breads with improved texture, cohesiveness, and overall quality, offering a promising pathway for healthier and more acceptable gluten‐free baked products (Diowksz and Sadowska 2021).

It is hypothesized that the incorporation of whole flours from forage palm, buckwheat, and teff enhances both the technological performance and sensory acceptance of gluten‐free breads, while contributing to improved nutritional quality. Accordingly, this study characterized the proximate composition and water‐holding capacity of these flours, applied them in bread formulations, and employed mathematical modeling to optimize the process. The optimized breads were then evaluated for physicochemical, nutritional, and sensory attributes.

## 2 Materials and Methods

### 2.1 Material

Tertiary and quaternary cladodes of forage palm (*O. ficus‐indica*) were harvested in March 2023 in Couto Magalhães de Minas, Minas Gerais, Brazil (18°04ʹ44ʺ S, 43°28ʹ40ʺ W), at an altitude of 710 m. The research was registered in the National System for the Management of Genetic Heritage and Associated Traditional Knowledge (SisGen) under registration number AF2C488. The choice of the main flours used in this study was based on their nutritional and technological potential. Buckwheat, a pseudocereal, is recognized for its high‐quality protein, favorable amino acid profile, and richness in micronutrients, while teff, a gluten‐free cereal, is valued for its mineral content, dietary fiber, and balanced protein composition (Brites et al. 2022; Gebru et al. 2020). Forage palm, in turn, was selected due to its innovative character in gluten‐free formulations, as it provides mucilage polysaccharides with high water‐holding capacity that enhance batter hydration and texture, in addition to offering dietary fiber and bioactive compounds of nutritional relevance (da Silva et al. 2024).

The detailed formulation of the bread samples is provided in Table S1. Enzymes were included as technological aids to improve batter performance: TGase promotes protein cross‐linking and strengthens the structural matrix; xylanase acts on hemicelluloses, improving batter extensibility and volume; phospholipase modifies lipid–protein interactions, contributing to better crumb structure and softness; and α‐amylase hydrolyzes starch, increasing fermentable sugars and enhancing loaf volume and crust color (Mohammadi et al. 2022).

In this study, the enzymes employed were TGase from *Streptomyces mobaraensis* (100 ppm), xylanase (bacterial hemicellulose, Megacell 899 C, 30 ppm), phospholipase (Megazyn 10260, 30 ppm), and α‐amylase from *Aspergillus oryzae* (10 ppm). All chemical reagents used in the analysis of the final products were of analytical grade and conformed to the purity standards required by the applied methodologies. For the analyses performed on both flours and bread samples, the following chemical reagents of analytical grade were employed: for protein determination, H_2_SO_4_, HCl, NaOH, copper sulfate, potassium sulfate, and boric acid; for lipid analysis, chloroform, methanol, and sodium sulfate; for soluble and insoluble dietary fiber, α‐amylase, protease, amyloglucosidase, ethanol, acetone, Celite, monosodium phosphate monohydrate, and anhydrous disodium phosphate; for total soluble phenolic compounds, Folin–Ciocalteu reagent, sodium carbonate, and gallic acid; and for oxygen radical absorbance capacity (ORAC), 6‐hydroxy‐2,5,7,8‐tetramethylchroman‐2‐carboxylic acid (Trolox), fluorescein, and 2,2′‐azobis(2‐methylpropionamidine) dihydrochloride (AAPH).

### 2.2 Obtention of Whole Flour From Forage Palm Cladodes

After collecting, the cladodes were washed with potable water to remove surface impurities, sanitized in a sodium hypochlorite solution (200 ppm) for 30 min, and rinsed with potable water to eliminate chlorine residues. Glochides (spines) were manually removed using knives, and the cladodes were packed in high‐density polyethylene bags and stored in a horizontal freezer CHA31CBNNA90 (Consul, Joinville, SC, Brazil) at −18°C (da Silva et al. 2024).

For the drying process, the samples were thawed overnight in a BF Inox 2520 industrial refrigerator (BF Cozinhas, São Paulo, Brazil), cut into cubes with approximate dimensions of 130 × 130 × 100 mm, and distributed on trays (0.1225 m^2^) for dehydration in a Frugal20 dehydrator (Slow Juicer Brasil, Joinville, Brazil) with a nominal power of 2000 W, operated at a controlled temperature of 60°C for 5 h until reaching a moisture content of less than 15%. The dehydrated cubes were subsequently milled in a TE‐350 ball mill (Tecnal, Piracicaba, Brazil) until a particle size smaller than 350 µm was obtained.

The resulting forage palm whole flour was packed in biaxially oriented polypropylene bags coated with aluminum foil for light protection and stored in a freezer (DFN41; Electrolux, Curitiba, Brazil) at −18°C to preserve its physicochemical properties.

### 2.3 Proximate Composition of Whole Flours

The proximate composition of the whole flours was determined through the analysis of moisture content (method 44‐17.01), ash content (method 08‐01.01), and protein content (method 46‐12.01; *N* = 6.25), according to the procedures established by the American Association of Cereal Chemists International (AACCI 2010). The lipid content was determined following the method described by Bligh and Dyer (1959). The total carbohydrate content was calculated by difference. All analyses were conducted in triplicate, and the results were expressed as percentages.

### 2.4 Experimental Design

Response surface methodology was employed using a simplex‐centroid mixture design with constraints (Table 1) to evaluate the flours in the development of gluten‐free breads, as described by Rodrigues and Iemma (2014). Buckwheat whole flour (x_1_), teff whole flour (x_2_), and forage palm whole flour (x_3_) were utilized as the independent variables. A constraint was applied to the experimental design, limiting the maximum concentration of forage palm whole flour to 10% (w/w in db.) of the flour mixture, due to its high water absorption capacity (WAC). To address this limitation, the formulation incorporated buckwheat whole flour as a compensatory component, given its broader commercial availability and established role as a reliable raw material in gluten‐free product development.

**TABLE 1:** Codified and actual levels of the simplex‐centroid mixture design with constraints for the development of gluten‐free breads with whole flours of buckwheat, teff, and forage palm.

Trial	Coded level			Real level		
	x_1_	x_2_	x_3_	X_1_	X_2_	X_3_
1	1.000	0.000	0.000	100	0	0
2	0.000	1.000	0.000	0	100	0
3	0.900	0.000	0.100	90	0	10
4	0.500	0.500	0.000	50	50	0
5	0.950	0.000	0.050	95	0	5
6	0.450	0.500	0.050	45	50	5
7	0.817	0.167	0.017	81.7	16.7	1.7
8	0.317	0.667	0.017	31.7	66.7	1.7
9	0.767	0.167	0.067	76.7	16.7	6.7
10	0.633	0.333	0.033	63.3	33.3	3.3
11	0.633	0.333	0.033	63.3	33.3	3.3
12	0.633	0.333	0.033	63.3	33.3	3.3
13	0.633	0.333	0.033	63.3	33.3	3.3

*Note*: x_1_ and X_1_ denote buckwheat whole flour; x_2_ and X_2_ denote teff whole flour; and x_3_ and X_3_ denote forage palm whole flour, corresponding respectively to coded and real levels.

### 2.5 WAC of the Flour Mixtures

The WAC was determined according to the methodology of Schmiele et al. (2013), as it is a key functional property influencing batter hydration, structure formation, and final bread quality in gluten‐free formulations (Šmídová and Rysová 2022). For this, 1 g of the sample (on a dry weight basis) was added to pre‐weighed 15‐mL Falcon tubes, along with 9 mL of distilled water. The samples were maintained at room temperature for 30 min and manually shaken intermittently every 5 min. Subsequently, the tubes were centrifuged at 3200 × *g* for 10 min in a Fanem Baby Centrifuge (Tecnal, Piracicaba, Brazil). The supernatant was transferred to pre‐weighed Petri dishes, and the liquid was evaporated in a TE−349/1 oven (Tecnal, Piracicaba, Brazil) at 105°C, with air renewal and forced circulation (1 m·s^−1^). The residue retained in the tube was weighed, and the WAC was calculated using Equation (1):

(1) $$\mathrm{WAC}=\left(w_{f}-w_{i}\right)/\left(w_{i}-s_{f}\right),$$

where WAC = water absorption capacity (g of water per g of sample in dry weight); *w*_i_ = initial weight of sample (g); *w*_f_ = final weight of hydrated sample (g); and *s*_f_ = soluble fraction (g).

### 2.6 Preparation of the Batters and Gluten‐Free Breads

The preparation of the gluten‐free batters involved weighing the ingredients (whole flours of buckwheat, teff, and forage palm, carboxymethylcellulose, egg albumin, iodized kitchen salt, sucrose, instant dry yeast, water, and palm fat) and technological aids (enzymes). The dry ingredients and technological aids were added to a PHP500 turbo mixer (Philco, Manaus, Brazil), and water was gradually incorporated. The mixture was beaten with a coated flat beater at maximum speed for 4 min to ensure batter homogenization. At this stage, three portions of 50 g of batter were collected for specific gravity and texture analysis. The remaining batter was divided into portions of 65 g and placed in pre‐greased rectangular steel Flander molds (100 × 55 × 30 mm) with a commercial release agent containing water, oil, and emulsifier. The batter was manually leveled using a silicone spatula.

The molds with the batters were fermented in a Compacta fermenter (Prática, Pouso Alegre, Brazil) at 33°C and 80% relative humidity for 50 min. After proofing, the molds were transferred to an FC4EMV convection electric oven (Venâncio, Caxias do Sul, Brazil), preheated to 160°C, and baked for 25 min. Upon complete baking, the loaves were removed from the molds, cooled to room temperature, and stored in low‐density polyethylene bags at room temperature.

### 2.7 Technological Aspects and Physicochemical Characteristics of the Batters

#### 2.7.1 Specific Gravity

The specific gravity of the batters was determined in triplicate, according to method 55‐50.01 of the AACCI (2010). The results were reported in grams per cubic centimeter.

#### 2.7.2 pH

The pH of the batters was determined using method 981.12 of the Association of Official Analytical Chemists (AOAC 2019), with a mPA210 bench potentiometer (Tecnopon, Piracicaba, Brazil). The analysis was conducted in triplicate.

#### 2.7.3 Texture Profile

The instrumental texture of the batters was evaluated according to method 74‐09.01 of the AACCI (2010), using a TAXT Plus texture analyzer (Stable Micro Systems, Godalming, England). The equipment was configured with a P/0.5S probe (5 mm diameter) and an HDP/90 platform in compression force mode, with pretest, test, and posttest speeds set at 0.5 mm/s, a time interval of 1 s between cycles, 50% penetration of the total distance, and a force threshold of 0.025 N. These settings were established based on preliminary tests carried out to ensure appropriate conditions for the analysis. The dependent variables obtained from this analysis were batter firmness (N), consistency (N·s), adhesiveness (N), and viscosity index (N·s).

### 2.8 Technological Aspects and Physicochemical Characteristics of the Breads

#### 2.8.1 Specific Volume

The specific volume of the loaves was determined by the millet seed displacement method, following method 10‐05.01 of the AACCI (2010). The analysis was performed in triplicate, and the results were expressed as cubic centimeters per gram.

#### 2.8.2 Browning Index, Whiteness Index, and Color Index of the Loaves

The browning index, whiteness index, and color index of the breads were determined based on the L*, a*, and b* instrumental color parameters, obtained according to method 14‐22.01 of the AACCI (2010), using a CM‐5 spectrophotometer (Konica Minolta, Tokyo, Japan). From these values, the browning index (Equation 2) for the crust was calculated according to the methodology proposed by Sharanagat and Nema (2023), whereas the whiteness index (Equation 3) and the color index (Equation 4) for the crumb were calculated as described by Sharanagat and Nema (2023) and Choque‐Quispe et al. (2022), respectively.

(2) $$\begin{array}{ccc} \mathrm{Browning}\;\mathrm{index} & = & \left[\left\{\left(\frac{a^{*}+1.75L^{*}}{5.64a^{*}+a^{*}-3.01b^{*}}\right)-0.31\right\}\times 100\right]\\ & & \times \;\frac{1}{0.17}, \end{array}$$

(3) $$\mathrm{Whiteness}\;\mathrm{index}=100-\sqrt{{\left(100-L^{*}\right)}^{2}+{a^{*}}^{2}+},$$

(4) $$\mathrm{Color}\;\mathrm{index}=\frac{a^{*}\times 1000}{L^{*}\times a^{*}},$$

where (i) if color index is between −40 and −20, the colors range from blue‐violet to deep green; (ii) if color index is between −20 and −2, the colors range from deep green to yellowish green; (iii) if color index is between −2 and +2, it represents yellowish green; (iv) if color index is between +2 and +20, the colors range from light yellow to deep orange; and (v) if color index is between +20 and +40, the colors range from deep orange to deep red.

#### 2.8.3 Evaluation of the Crumb Texture Profile

The texture profile of the crumb was evaluated according to method 74‐09.01 of the AACCI (2010), using a TA‐XT Plus texture analyzer (Stable Micro Systems, Godalming, England). The equipment was configured with a P36 probe (28 mm height) and an HDP/90 platform in compression force mode. The pretest, test, and posttest speeds were set at 1.00, 1.00, and 10 mm·s^−1^, respectively. The time between cycles was set at 1 s, with a penetration depth of 40% of the total distance and a force threshold of 0.025 N. For the analysis, the loaves were sliced into 12‐mm‐thick slices using an FPV12 slicer (Venâncio, Venâncio Aires, Brazil). After slicing, two slices were overlapped and cut using a round mold with a 30‐mm diameter. Each test was performed 10 times. The variables analyzed included crumb firmness (N), hardness (N), springiness, cohesiveness, gumminess (N), chewiness (N), and resilience.

### 2.9 Numerical Optimization and Validation of the Mathematical Model

The optimization of the gluten‐free bread production process was conducted following the methodology proposed by Derringer and Suich (1980). The independent variables were optimized within the studied range. Statistically significant dependent variables were set as maximum, minimum, or in range values (according to the desired effect), with each response assigned a level of importance (where 1 represented the lowest importance and 5 the highest). The optimal points were performed in true triplicate to validate the predictions of the mathematical models.

### 2.10 Proximate Analysis and Total Calorie Value of the Optimized Formulations

Proximate analysis was conducted on the optimized bread samples to determine their moisture content (method 44‐15.02), protein content (method 46‐13.01; nitrogen factor = 6.25), ash content (method 08‐01.01), and total dietary fiber content (method 32‐07.01) according to the methods of the AACCI (2010). The lipid content was assessed using the method described by Bligh and Dyer (1959), and digestible carbohydrate content (sugars and digestible starch) was calculated by difference. All analyses were performed in triplicate, and the results were expressed as grams per 100 grams (wet basis). The total caloric value was calculated using the Atwater conversion method, as described by De Souza and Schmiele (2021).

### 2.11 Bioactive Compounds and Antioxidant Activity

#### 2.11.1 Extraction of Total Soluble Phenolic Compounds

Buckwheat, teff, and forage palm contain considerable amounts of soluble phenolic compounds, especially when compared to other flours commonly used in gluten‐free breadmaking. In this context, the extraction of total soluble phenolic compounds was performed as described by Costantini et al. (2014), with modifications. A 1‐g portion of the sample (in triplicate) was weighed and transferred into 15‐mL Falcon tubes, to which 4.7 mL of an 80% (v/v) hydroethanolic extracting solution, as described by Brites et al. (2022), was added. The samples were homogenized by vortex agitation to ensure uniform dispersion of the components within the extracting solution. Extraction was subsequently assisted by low‐frequency ultrasound (40 kHz, 100 W), employing two 30‐min cycles with a 20‐min rest interval between extractions. All procedures were conducted at room temperature (∼20°C). Following the extraction, the tubes were centrifuged at 5000 × *g* for 10 min at 20°C using a SL‐5GR centrifuge (Spinlab, Ribeirão Preto, Brazil). The resulting supernatant was then transferred to a volumetric flask, and the final volume was adjusted to 10 mL using the same extracting solution.

#### 2.11.2 Quantification of Total Soluble Phenolic Compounds

The determination of total soluble phenolic compounds was performed using the Folin–Ciocalteu method. For this analysis, 100 µL of the extract was combined with 250 µL of 0.2 N Folin–Ciocalteu reagent, 3 mL of distilled water, and 1 mL of a 15% (w/v) sodium carbonate (Na_2_CO_3_) solution. The reaction mixture was incubated in the dark for 30 min to allow complete color development. Absorbance was measured at 750 nm using a SpectraMax Paradigm microplate spectrophotometer (Molecular Devices, San José, USA). An 8‐point calibration curve was constructed with gallic acid standards at concentrations ranging from 0 to 600 mg·L^−1^ (*y* = 0.0009*x* + 0.0505; *r* = 0.9938). All measurements were conducted in quadruplicate, and the results were expressed as milligrams of gallic acid equivalents (GAE) per 100 grams of sample on a wet basis.

#### 2.11.3 ORAC

The antioxidant capacity of the extracts was assessed using the ORAC method, as described by Dávalos et al. (2004), with modifications introduced by Camponogara et al. (2024). The assay was conducted in a black 96‐well microplate. Initially, 20 µL of either the sample extracts or a standard solution of 6‐hydroxy‐2,5,7,8‐tetramethylchroman‐2‐carboxylic acid (Trolox), prepared at concentrations ranging from 0 to 0.75 mM Trolox·mL^−1^, was transferred into the wells. Subsequently, 120 µL of a 70‐nM fluorescein solution was added, and the plate was incubated at 37°C for 15 min. Following incubation, 200 µL of a 12‐mM solution of AAPH was added to initiate the oxidative reaction.

Fluorescence decay was monitored every minute for 80 min using a Spectra‐Max Paradigm microplate spectrophotometer (Molecular Devices, San José, USA), with excitation and emission wavelengths set at 485 and 525 nm, respectively. The microplate was maintained at 37°C, and orbital shaking was applied for 5 s before each reading. A 6‐point standard calibration curve was constructed using Trolox concentrations ranging from 0 to 0.75 mM Trolox·L^−1^ (*y* = 9.1791*x* + 3.5188; *r* = 0.9955). The analysis was performed in triplicate, and the results were expressed as millimoles of Trolox equivalents (TE) per 100 grams of sample on a wet basis.

### 2.12 Sensory Analysis of the Optimized Formulations

Due to the limited availability of gluten‐free products in the local market (city population ∼50,000 inhabitants), it was not possible to recruit a representative pool of habitual gluten‐free consumers. Therefore, a convenience sample primarily composed of college students was employed, as they represent an accessible group of potential consumers of innovative bakery products. The sensory analysis was conducted in two sessions. In Session 1 (Focus Group), 23 panelists were divided into four groups, while in Session 2, 94 untrained panelists participated (Acceptance Test employing the Hedonic Scale, Purchase Intention assessment, and Check All That Apply—CATA). Informed consent was obtained from all participants, who signed the consent forms before participation. The study was conducted following the Declaration of Helsinki and approved by the Research Ethics Committee of the Federal University of Jequitinhonha and Mucuri Valleys (Certificate of Ethical Appreciation Presentation number: 89302718.7.0000.5108), approved on June 18, 2018, with the term extended on October 7, 2022.

The recruitment of panelists for the gluten‐free bread Focus Group was conducted through an online form. During the first session, four groups were evaluated at different time intervals. Samples were presented to each group, and the collection of sensory descriptors was facilitated by a moderator, with assistance from support staff for term recording, over an approximate duration of 40 min per group. Upon completion of the sessions, 19 sensory descriptors were selected for the breads.

In the second session, the samples were presented monadically, and Acceptance (Hedonic Scale), Purchase Intention, and CATA tests were performed for each sample. Participants were instructed to evaluate the acceptance of attributes (overall impression, aroma, appearance, color, texture, and flavor) using a 9‐point hedonic scale ranging from 1 = *Dislike extremely* to 9 = *Like extremely*. Purchase Intention was assessed using a 5‐point scale ranging from 1 = *I would definitely not buy this product* to 5 = *I would definitely buy this product*.

The CATA evaluation form was developed based on the 19 sensory descriptors defined during the Focus Group, and panelists were instructed to select all terms that best described each sample (Stone et al. 2020).

### 2.13 Statistical Analysis

Proximate composition data were subjected to analysis of variance (ANOVA), considering a significance level of 5%. When statistical differences were identified, Tukey's test was applied for means comparison. The data related to mass and bread evaluation were analyzed using response surface methodology, employing a constrained simplex‐centroid mixture design, following Equation (5) described by Rodrigues and Iemma (2014). The regression coefficients and ANOVA were calculated using a significance level of 10%, and models presenting a minimum coefficient of determination (*R* ^2^) of 80% were considered acceptable.

(5) $$Y=\beta_{1x1}+\beta_{2x2}+\beta_{3x3}+\beta_{12x1x2}+\beta_{13x1x3}+\beta_{23x2x3}+\beta_{123x1x2x3}+\varepsilon ,$$

where *Y* represents the response variable under study; *β_i_* and *β_ij_* are the regression coefficients for the pseudocomponents and for the binary and ternary interactions, respectively; x*i* and x*j* are the coded values of the independent variables; and *ε* corresponds to the experimental error.

For the data related to the optimized formulations, total soluble phenolic compounds, and antioxidant capacity, the Student's *t*‐test was applied, adopting a significance level of *p* < 0.05. Terms identified by the Focus Group were analyzed based on their frequency and relevance, in consultation with the researchers. Acceptance data obtained through the hedonic scale were tested for normality using the Shapiro–Wilk test and for homogeneity of variance using Levene's test (RStudio). As the assumptions for parametric testing were not satisfied, the data were analyzed using the nonparametric Mann–Whitney–Wilcoxon test at a 5% significance level, and the results were presented as box plots (RStudio). Purchase intent data were represented using histograms. Results from the CATA test were analyzed using the chi‐square test (RStudio) and visualized through radar charts (RStudio).

## 3 Results and Discussion

### 3.1 Proximate Composition of Flours

The proximate composition analysis revealed distinct characteristics among forage palm, buckwheat, and teff whole flours. These characteristics may provide a variety of nutritional options, aligning with different dietary needs and offering diverse health benefits. The results related to the proximate composition of the flours are presented in Table 2.

**TABLE 2:** Proximate composition of forage palm, buckwheat, and teff whole flours (g·100 g^−1^, wet basis).

Component	Forage palm	Buckwheat	Teff
Moisture	12.68 ± 0.13^a^	10.01 ± 0.31^b^	9.43 ± 0.01^b^
Lipids	2.75 ± 0.10^ns^	2.67 ± 0.03^ns^	1.83 ± 0.11^ns^
Proteins	10.24 ± 0.06^b^	11.58 ± 0.01^a^	9.76 ± 0.02^b^
Ashes	17.92 ± 0.09^a^	1.99 ± 0.01^b^	1.92 ± 0.02^b^
Total carbohydrates^a^	56.41 ± 0.20	73.75 ± 0.31	77.06 ± 0.11

*Note*: Values correspond to the arithmetic mean of three replicates ± standard deviation. Means followed by different letters in the same row indicate statistically significant differences according to Tukey's test (*p* < 0.05).

^a^ Standard deviation calculated by error propagation.

Abbreviation: ns, not significant.

Moisture content exhibited significant variation among the analyzed flours, with the highest value observed in forage palm whole flour (12.68 g·100 g^−1^), followed by buckwheat whole flour (10.01 g·100 g^−1^) and teff whole flour (9.43 g·100 g^−1^), which exhibited the lowest moisture content. This variation can be attributed to differences in processing methods and drying times, which directly influence the moisture levels of the flours. However, it is noteworthy that all obtained values remain within the maximum moisture content limit of 15% (w/w) set by Brazilian legislation for plant‐based flours (Célia et al. 2022). Therefore, the flours analyzed in this study comply with current regulatory standards, ensuring proper storage conditions and maintaining the safety and quality required for commercialization.

The lipid content was relatively similar across the three flours (*p* > 0.05)—forage palm whole flour (2.75 g·100 g^−1^), buckwheat (2.67 g·100 g^−1^), and teff (1.83 g·100 g^−1^), indicating a general similarity in lipid content across the flours. These findings align with the results reported by Al‐Marazeeq et al. (2023), which documented a lipid content of 2.1 g·100 g^−1^ in forage palm whole flour. The variation in lipid content among the flours may be influenced by several factors, including plant genotype, cultivation conditions, seed maturation stage, and processing methods, which can contribute to minor fluctuations in fat content across different flour samples, even when derived from the same plant species (Saldanha et al. 2023).

Buckwheat whole flour exhibited the highest protein content (11.58 g·100 g^−1^), followed by forage palm (10.24 g·100 g^−1^) and teff (9.67 g·100 g^−1^). This protein content is consistent with expectations, as one of the primary attributes of buckwheat is its high protein level, as highlighted by Bhinder et al. (2022) and Shreeja et al. (2021). Buckwheat is not only notable for its protein content but also for the superior quality of its proteins, which are characterized by a balanced composition of essential amino acids, including lysine, methionine, cysteine, and tryptophan, which are vital for various biological and metabolic functions (Luthar et al. 2020). Furthermore, buckwheat proteins exhibit significant functional advantages, such as enhanced water retention capacity, foam formation, and emulsifying properties, compared to proteins from other grains (cereals and pseudocereals) (Bhinder et al. 2022). These attributes make buckwheat an excellent candidate for the development of gluten‐free breads, where it has the potential to improve texture, moisture retention, and overall product quality.

Minerals are essential for the proper functioning of the human and animal body, and constitute approximately 4% of total body mass. Like vitamins, minerals cannot be synthesized endogenously and must be acquired through dietary intake (Matthewman and Costa‐Pinto 2023). Mineral content varied considerably among the flours. Forage palm whole flour exhibited an exceptionally high ash content (17.92 g·100 g^−1^), while buckwheat (1.99 g·100 g^−1^) and teff (1.92 g·100 g^−1^) flours presented significantly lower and similar values. Similar results were reported by da Silva et al. (2024), who found an ash content of 15.13 g·100 g^−1^ in forage palm whole flour (on a dry basis).

The highest total carbohydrate content was observed in teff whole flour (77.06 g·100 g^−1^), followed by buckwheat (73.75 g·100 g^−1^) and forage palm (56.41 g·100 g^−1^). In teff, the starch content has been reported to reach approximately 72 g·100 g^−1^, accompanied by around 3 g·100 g^−1^ of insoluble dietary fiber and 5 g·100 g^−1^ of soluble dietary fiber. In the case of buckwheat, the carbohydrate fraction has been shown to comprise approximately 70 g·100 g^−1^ of starch (Zamaratskaia et al. 2024) and approximately 12 g·100 g^−1^ of total dietary fiber, of which 52% is insoluble and 48% is soluble (D'Amaro et al. 2024). The soluble fiber fraction has been characterized by a high content of pectins, arabinogalactans, and xyloglucans (Zamaratskaia et al. 2024). In contrast, the carbohydrate composition of forage palm whole flour has been found to consist almost entirely of dietary fiber (∼67 g·100 g^−1^), with an approximately equal distribution between the soluble and insoluble fractions (da Silva et al. 2024; Shoukat et al. 2023; Di Bella et al. 2022).

### 3.2 WAC of the Flour Blends

**FIGURE 1:**
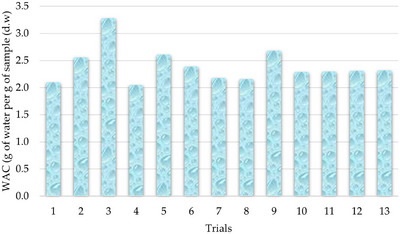
Water absorption capacity (WAC) of the flour blends.

The WAC is defined as the amount of water retained per gram of sample and is regarded as a critical parameter in the technofunctional characterization of flours. This property is intrinsically linked to the reconstitution potential of dry ingredients and exerts a significant influence on both the quality and yield of baked products, particularly in gluten‐free formulations (Okwunodulu et al. 2024). The values obtained for the WAC of the evaluated flour blends are presented in Figure 1.

According to Djeghim et al. (2021), the WAC of whole flours used individually or in blends can be influenced by multiple factors, including pH, the presence of hydrophilic components, and flour porosity. Additionally, particle size, as well as the quantity and molecular structure of macronutrients, particularly starch and protein, exerts significant effects on this property. In the present study, WAC values obtained in the experimental trials ranged from 2.05 to 3.28 g of water per gram of sample on a dry basis. The results were explained at 98.74% by the fitted mathematical model (Equation 6), which was deemed predictive. The ANOVA yielded a *F*_calc_/*F*_tab (4;8;0.05)_ ratio of 48.47 and a *p*‐value of <0.001.

(6) $$\mathrm{WAC}=2.08x_{1}+2.53x_{2}+3.26x_{3}-1.03x_{12}-2.04x_{2}x_{3},$$

where WAC = water absorption capacity (g of water per g of sample, dry basis); x_1_ = buckwheat whole flour; x_2_ = teff whole flour; and x_3_ = forage palm whole flour.

**FIGURE 2:**
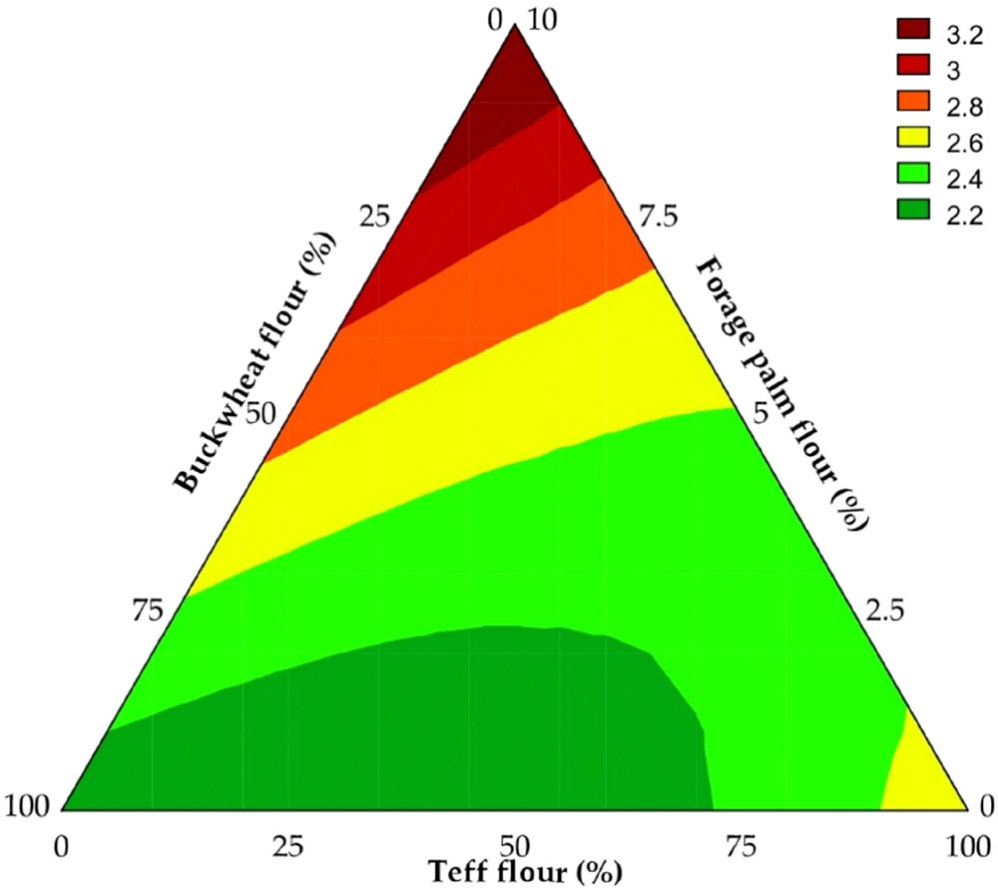
Contour plot illustrates the predicted water absorption capacity of flour blends employed in the development of gluten‐free bread formulations.

As illustrated in Figure 2, the highest WAC value was recorded with the greatest level of forage palm whole flour incorporation (*β*_3_ = 3.26, *p* < 0.001), followed by teff whole flour (*β*_2_ = 2.53, *p* < 0.001) and buckwheat whole flour (*β*_1_ = 2.08, *p* < 0.001). A negative interaction effect on WAC was observed when buckwheat whole flour was combined with teff whole flour (*β*_12_ = –1.03, *p* < 0.001). This reduction was even more pronounced when buckwheat flour was blended with forage palm flour (*β*_23_ = –2.04, *p* < 0.001).

**FIGURE 3:**
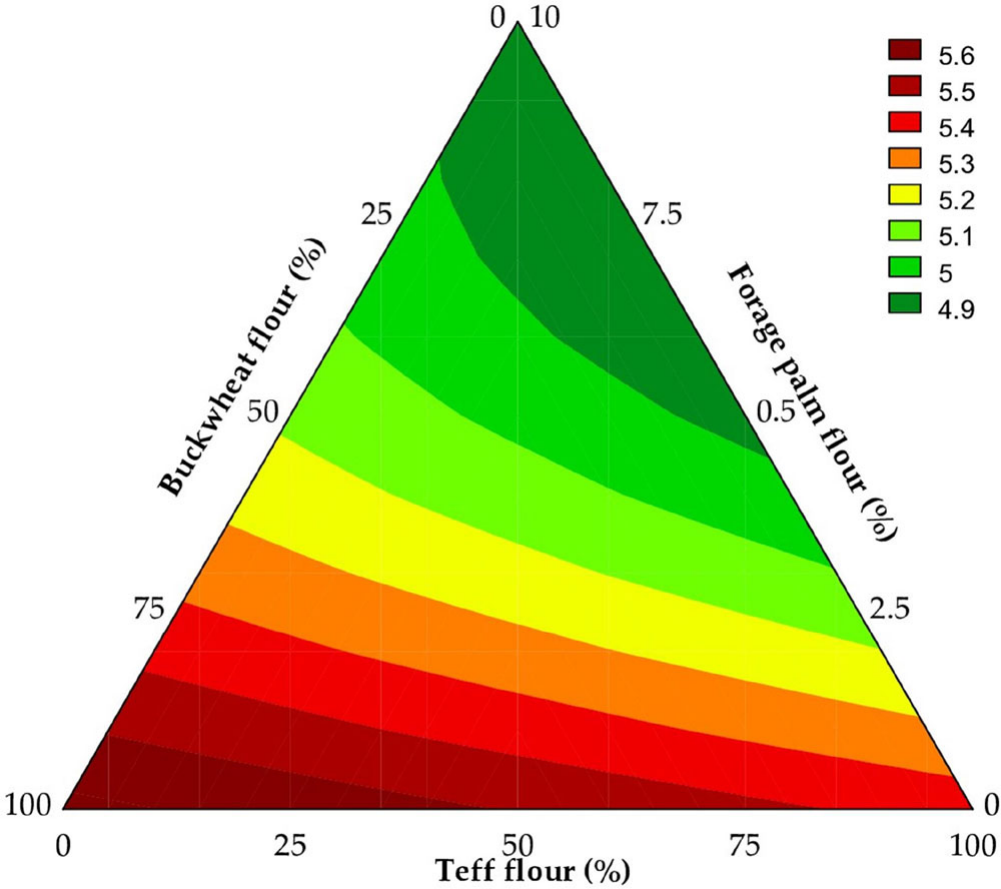
Contour plot illustrates the predicted pH of the batters employed in developing gluten‐free bread formulations.

The positive contribution of forage palm whole flour to the WAC of the blends has been attributed to the high hydration capacity of its dietary fibers, particularly those associated with mucilage. According to de Andrade Alves et al. (2021), the dietary fibers present in forage palm enhance water absorption by promoting hydrogen bond formation between the functional hydroxyl (─OH) groups of biopolymers and water molecules. Although teff and buckwheat whole flours demonstrated less pronounced effects, their capacity to absorb water has been documented in several studies, supporting their technological feasibility in the development of gluten‐free baked products (Vicente et al. 2024).

### 3.3 Physicochemical and Technological Aspects of the Batters

The results obtained from the pH, specific gravity, and instrumental texture analyses of the batters formulated for gluten‐free bread are presented in Table 3.

**TABLE 3:** Specific gravity, pH, and instrumental texture of the batters for preparing gluten‐free breads using whole flours from forage palm, buckwheat, and teff.

Trial	pH	Specific gravimetry (g·cm^−3^)	Firmness (N)	Consistency (N·s)	Adhesiveness (N·s)	Viscosity index (N·s)
1	5.62 ± 0.05	0.98 ± 0.02	0.87 ± 0.02	21.28 ± 0.74	−0.53 ± 0.01	−9.18 ± 0.99
2	5.35 ± 0.02	1.02 ± 0.01	0.07 ± 0.01	1.57 ± 0.08	−0.02 ± 0.01	−0.07 ± 0.03
3	4.86 ± 0.01	0.93 ± 0.02	0.40 ± 0.01	9.75 ± 0.31	−0.24 ± 0.01	−7.67 ± 0.27
4	5.54 ± 0.02	1.09 ± 0.02	0.21 ± 0.01	5.31 ± 0.34	−0.11 ± 0.01	−1.95 ± 0.21
5	5.06 ± 0.01	0.97 ± 0.01	0.67 ± 0.06	18.01 ± 0.97	−0.43 ± 0.07	−7.80 ± 4.28
6	4.85 ± 0.02	1.05 ± 0.01	0.26 ± 0.01	6.52 ± 0.20	−0.15 ± 0.01	−2.96 ± 0.14
7	5.31 ± 0.02	0.97 ± 0.01	0.49 ± 0.01	12.05 ± 0.36	−0.30 ± 0.01	−9.93 ± 2.36
8	5.14 ± 0.01	1.03 ± 0.01	0.16 ± 0.01	3.77 ± 0.26	−0.08 ± 0.01	−1.30 ± 0.17
9	4.89 ± 0.01	1.02 ± 0.01	0.41 ± 0.01	10.41 ± 0.23	−0.23 ± 0.01	−6.76 ± 0.84
10	5.07 ± 0.03	1.07 ± 0.01	0.36 ± 0.01	9.16 ± 0.43	−0.21 ± 0.01	−4.12 ± 0.55
11	5.11 ± 0.02	0.97 ± 0.01	0.32 ± 0.01	8.00 ± 0.20	−0.18 ± 0.01	−3.63 ± 0.51
12	5.14 ± 0.02	1.06 ± 0.01	0.40 ± 0.01	9.93 ± 0.47	−0.23 ± 0.01	−5.97 ± 0.74
13	5.19 ± 0.01	1.01 ± 0.01	0.38 ± 0.01	9.04 ± 0.43	−0.21 ± 0.01	−4.38 ± 0.76

*Note*: Data are the means of three replicates ± standard deviation.

#### 3.3.1 pH

The pH values of the batters ranged from 4.85 to 5.62, with 97.35% of the variation explained by the predictive mathematical model (*F*_calc_/*F*_tab (4;8;0.05)_ = 19.20, *p* < 0.001) (Equation 7). As shown in Figure 3, it was observed that the interactions between forage palm whole flour and buckwheat whole flour (*β*_13_ = −0.66, *p* = 0.012), as well as between forage palm flour and teff whole flour (*β*_3_ = −0.96, *p* = 0.001), resulted in a significant reduction in pH compared to the isolated use of palm flour (*β*_3_ = 4.86, *p* < 0.001).

(7) $$\mathrm{pH}=5.63x_{1}+5.36x_{2}+4.86x_{3}-0.66x_{13}-0.96x_{2}x_{3},$$

where x_1_ = buckwheat whole flour; x_2_ = teff whole flour; and x_3_ = forage palm whole flour.

This behavior is attributed to the composition of forage palm, which is rich in acidic compounds, such as organic and phenolic acids, that contribute to the acidification of the system (da Silva et al. 2024). The presence of phenolic compounds in both buckwheat and teff also contributes to the acidification of the batter, as reported in other studies (Coţovanu et al. 2022; Xu et al. 2019). The reduction in pH directly affects the chemical equilibrium of the batter, potentially influencing protein solubilization, the stability of the structural network, and the interactions among formulation components, particularly those mediated by acid–base equilibria (Ramos Magalhães et al. 2023).

#### 3.3.2 Specific Gravity

The specific gravity is considered a pertinent parameter for assessing bread texture and quality, being inversely associated with the incorporation and retention of air within the batter. Higher values indicate reduced aeration and, consequently, a denser and firmer structure, whereas lower values indicate enhanced air retention, contributing to a lighter texture (Wang et al. 2025). Batter specific gravity values ranged from 0.93 to 1.09 g·cm^−^^3^. However, the addition of alternative flours did not influence this parameter, as indicated by the ANOVA (*R* ^2^ = 0.6308, *p* = 0.266), thereby precluding the development of contour plots for this response. However, a promising effect on air incorporation into the batter was observed with the addition of forage palm whole flour (*β*_1_ = 0.94, *p* < 0.001), followed by buckwheat whole flour (*β*_2_ = 0.94, *p* < 0.001) and teff whole flour (*β*_3_ = 1.01, *p* < 0.001).

#### 3.3.3 Instrumental Texture

**FIGURE 4:**
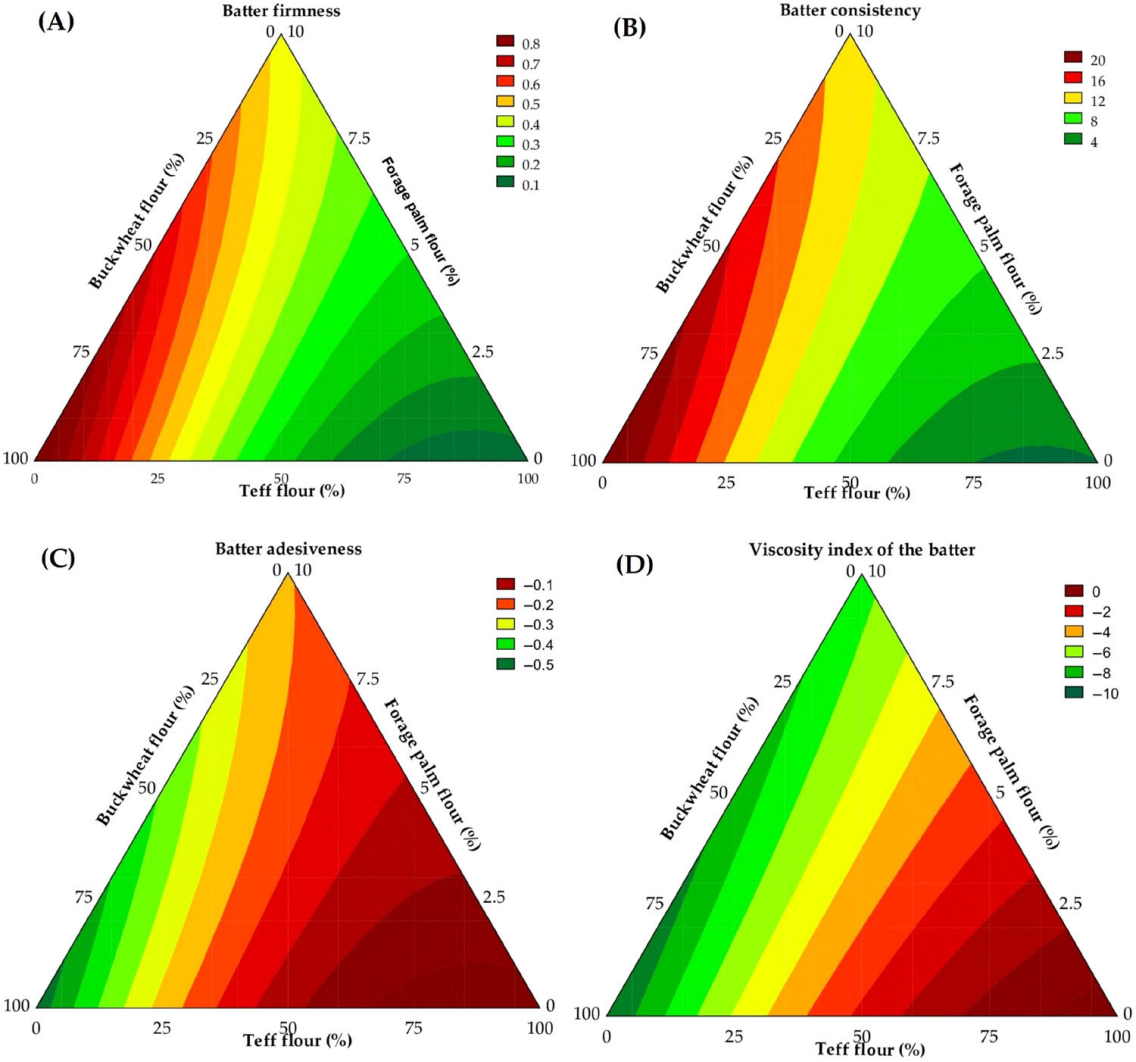
Contour plots illustrate the predicted instrumental texture of the batters employed in developing gluten‐free bread formulations.

Instrumental texture parameters facilitate the comprehension of batter behavior during processing and the quality of the final product (Burbano et al. 2022). The properties of firmness, consistency, and adhesiveness of the batters are closely correlated with the flour composition, particularly concerning the quantity and technofunctional properties of inherent proteins in the raw materials utilized. Consequently, batter firmness results (Table 3) ranged from 0.07 to 0.87 N, with 98.39% of the variability explained by the mathematical model (Equation 8) and ANOVA (*F*_calc_/*F*_tab (3;9;0.05)_ = 47.51, *p* < 0.001), thereby enabling the creation of a contour plot (Figure 4A).

(8) $$\mathrm{Firmness}\left(N\right)=0.863x_{1}+0.080x_{2}+0.430x_{3}-1.009x_{1}x_{2},$$

where x_1_ = buckwheat whole flour; x_2_ = teff whole flour; and x_3_ = forage palm whole flour.

The most notable increase in batter firmness was observed with higher additions of buckwheat whole flour (*β*_1_ = 0.863, *p* < 0.001). Conversely, the factor primarily contributing to reduced firmness was the interaction between buckwheat and teff whole flours (*β*_12_ = −1.009, *p* < 0.001). These findings suggest that a greater proportion of buckwheat promotes a firmer batter structure, likely due to its elevated insoluble fiber content, which enhances mechanical resistance (Gutierrez et al. 2023). In contrast, incorporating teff whole flour, characterized by a lower concentration of fibers, appears to mitigate this effect, resulting in a less rigid structure with diminished deformation resistance. This relationship between insoluble fiber content and batter texture was previously noted by Wang et al. (2021), who observed that the inclusion of such fibers enhances batter mechanical strength.

Higher batter consistencies are recognized to favor bread structure, volume, crumb texture, and overall acceptability (Aguiar et al. 2021). Batter consistency values ranged from 1.57 to 21.28 N·s, with 97.11% of the variability explained by the predictive mathematical model (Equation 9). The significance of the model was confirmed by the ANOVA (*F*_calc_/*F*_tab (3;9;0.05)_ = 26.17, *p* < 0.001), allowing for the generation of a contour plot (Figure 4B). The highest consistency was observed with the addition of buckwheat whole flour (*β*_1_ = 21.61, *p* < 0.001), whereas a significant reduction in this parameter was identified due to its binary interaction with teff whole flour (*β*_12_ = −25.73, *p* < 0.001).

(9) $$\mathrm{Consistency}\left(N\cdot s\right)=21.61x_{1}+1.88x_{2}+10.92x_{3}-25.73x_{1}x_{2},$$

where x_1_ = buckwheat whole flour; x_2_ = teff whole flour; and x_3_ = forage palm whole flour.

Adhesiveness is defined as the force required to detach the batter from a surface to which it adheres (Torres‐Pérez et al. 2024). Values for batter adhesiveness ranged from −0.53 to −0.02 N. The mathematical model (Equation 10) explained 98.28% of the observed variation, and the model's adequacy was supported by ANOVA (*F*_calc_/*F*_tab (3;9;0.05)_ = 44.38, *p* < 0.001). An increase in batter adhesiveness occurs by the interaction between buckwheat and teff whole flours (*β*_12_ = 0.67, *p* < 0.001), suggesting that this combination promotes a more cohesive and adhesive batter matrix, as observed in Figure 4C. This behavior may influence not only batter handling during processing but also technological attributes of the final bread, such as crumb structure and surface characteristics, which are directly perceived by consumers through visual appearance and tactile evaluation at the time of purchase.

(10) $$\begin{array}{ccc} \mathrm{Adhesiveness}\left(N\right) & = & -0.533x_{1}-0.029x_{2}-0.256x_{3}\\ & & +\;0.667x_{1}x_{2}, \end{array}$$

where x_1_ = buckwheat whole flour; x_2_ = teff whole flour; and x_3_ = forage palm whole flour.

Accordingly, adding buckwheat whole flour alone increases the batter firmness, hardness, and consistency, negatively impacting the final product's texture, as softer crumb structures are typically associated with lower values for these parameters. In contrast, the incorporation of teff whole flour exerted an opposing effect, contributing to batter softening. These contrasting effects may be attributed to the chemical composition of the flours. Buckwheat whole flour possesses high water absorption and retention capacities, which reduce batter malleability while enhancing firmness and consistency (Torbica et al. 2010). Conversely, the proteins in teff whole flour exhibit high solubility in aqueous media, favoring the development of a more flexible matrix and thereby reducing the batter's mechanical resistance.

Nevertheless, combining buckwheat and teff whole flours increases the batter adhesiveness, a phenomenon likely associated with weak interactions between the matrix components and water. Although the fibers and proteins in these flours interact with the aqueous phase, such interactions may not be sufficient to ensure efficient water binding within the matrix. Consequently, the batter surface becomes more adhesive, increasing its tendency to stick to processing equipment.

The absence of gluten in the formulations typically results in highly flowable batters that exhibit rheological behavior like cake batters and demonstrate reduced gas retention capacity compared to wheat‐based batters (Cappelli et al. 2020). In this context, enzymatic approaches such as the use of second‐generation lipases, which generate polar lipids with strong emulsifying capacity, could strengthen the batter structure by stabilizing gas cells and enhancing resistance to mechanical stress, thus mitigating the loss of functionality associated with gluten absence (Basit et al. 2025). Viscosity index values for the batters ranged from −9.93 to −0.07 N·s, with 86.62% of the variability explained by the predictive mathematical model (Equation 11). The model's significance was validated by ANOVA (*F*_calc_/*F*_tab (3;9;0.05)_ = 5.03, *p* < 0.001). The lowest viscosity values (Figure 4D) were associated with the addition of buckwheat whole flour (*β*_1_ = −10.02, *p* < 0.001) and forage palm whole flour (*β*_3_ = −7.42, *p* < 0.001). These findings suggest that incorporating these flours alters batter structure, rendering it less viscous and more suitable for machining, an attribute of relevance in developing gluten‐free baked products with defined textural and consistent characteristics.

(11) $$\mathrm{Viscosity}\;\mathrm{index}\left(N\cdot s\right)=-10.02x_{1}+0.55x_{2}-7.42x_{3}+7.88x_{1}x_{2},$$

where x_1_ = buckwheat whole flour; x_2_ = teff whole flour; and x_3_ = forage palm whole flour.

### 3.4 Physicochemical and Technological Aspects of Gluten‐Free Breads

#### 3.4.1 Browning Index, Whiteness Index, and Color Index of Gluten‐Free Breads

**FIGURE 5:**
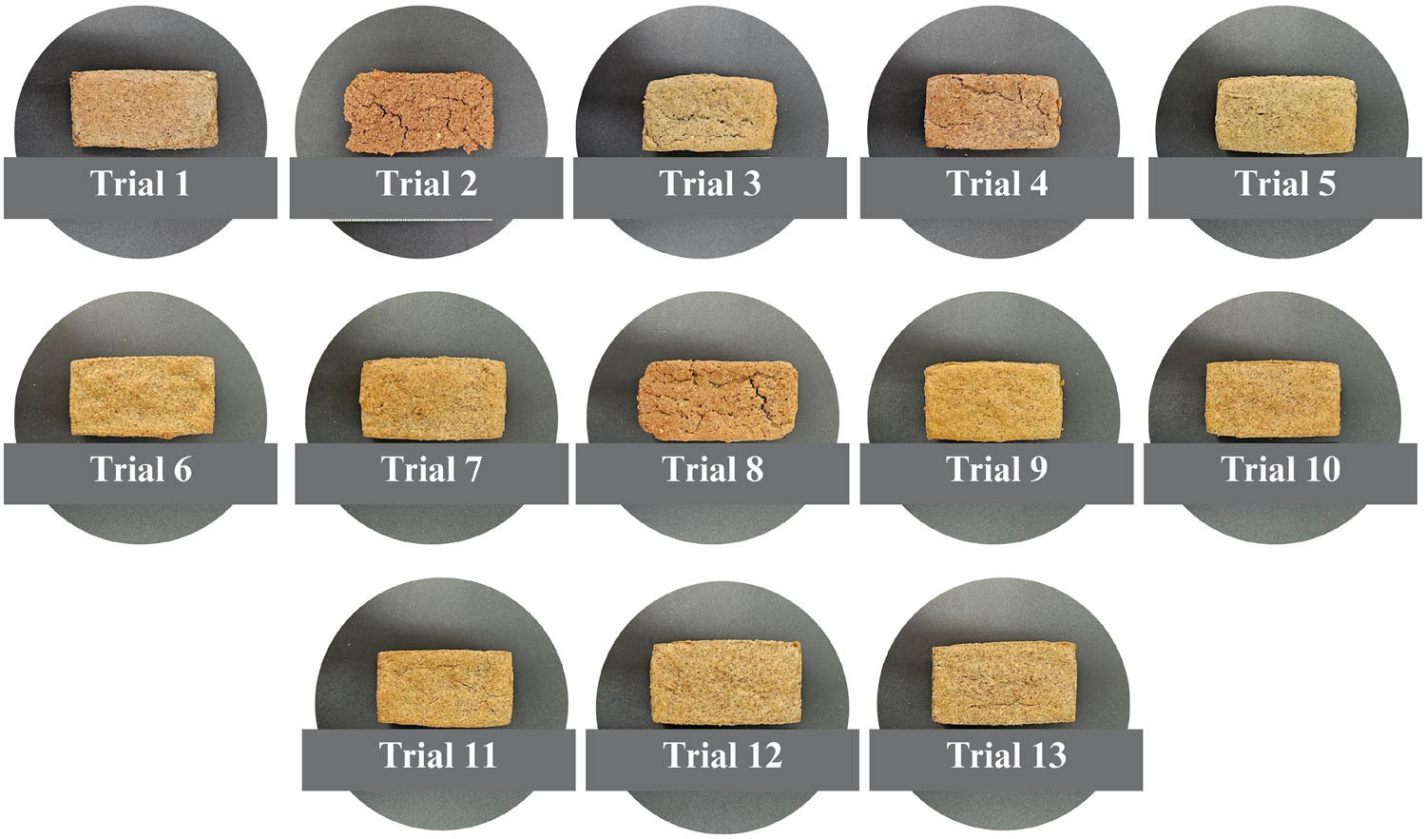
Visual appearance of the top of gluten‐free breads.

The data for the crust browning index, as well as the crumb whiteness and color index, are presented in Table 4. The browning index is recognized as a parameter indicative of the extent of nonenzymatic browning in each sample (Arora et al. 2021). In baked products, color development during the baking phase is considered critical and serves as an essential indicator for determining the endpoint of thermal processing. The brown coloration observed on the surface (Figure 5) is primarily attributed to melanoidins and other compounds produced through sugar caramelization. The addition of enzymes such as alpha‐amylase may enhance these reactions by hydrolyzing starch into reducing sugars, providing additional substrates for Maillard and caramelization reactions and potentially increasing the intensity of browning, which is particularly relevant because crust color is among the first and most important attributes evaluated by consumers at the point of purchase (Lohinova and Petrusha 2023; Güngör Ertuğral 2021).

**TABLE 4:** Experimental data regarding crust browning index, and crumb whiteness and color indexes of gluten‐free breads.

Trial	Browning index	Whiteness index	Color index
1	55.09 ± 5.44	41.88 ± 0.61	41.88 ± 0.61
2	68.33 ± 1.37	10.64 ± 0.29	10.64 ± 0.29
3	67.48 ± 2.63	33.29 ± 0.36	33.29 ± 0.36
4	64.51 ± 1.80	22.41 ± 0.34	22.41 ± 0.34
5	60.76 ± 4.44	41.02 ± 0.17	41.02 ± 0.17
6	73.93 ± 3.13	6.19 ± 0.17	6.19 ± 0.17
7	68.20 ± 4.51	36.86 ± 0.80	36.86 ± 0.80
8	68.06 ± 0.97	16.30 ± 0.69	16.30 ± 0.69
9	66.54 ± 3.14	40.27 ± 0.60	40.27 ± 0.60
10	66.93 ± 2.21	8.16 ± 0.39	8.16 ± 0.39
11	69.26 ± 2.21	37.77 ± 1.73	37.77 ± 1.73
12	63.91 ± 3.56	11.95 ± 1.66	11.95 ± 1.66
13	72.39 ± 3.33	41.08 ± 0.89	41.08 ± 0.89

*Note*: Data are the means of three replicates ± standard deviation.

As presented in Table 4, crust browning index values ranged from 55.09 to 73.93. Despite this observed variation, the independent variables under investigation did not exert a statistically significant effect on this parameter, as indicated by the coefficient of determination (*R* ^2^ = 70.23%) and the nonsignificant *p*‐value obtained from the ANOVA (*p* = 0.160). Consequently, developing a reliable mathematical model for predicting the behavior of the browning index was not feasible. Nevertheless, the results suggest that crust browning was primarily driven by nonenzymatic reactions occurring during the baking process (Figure 5). However, a darker crust coloration in gluten‐free breads was observed when teff whole flour (*β*_2_ = 67.62, *p* < 0.001) was used, whereas the incorporation of buckwheat whole flour (*β*_1_ = 56.81, *p* < 0.001) resulted in less intense brown shades. Additionally, the enzymes in the formulation—such as TGase, xylanase (bacterial hemicellulase), phospholipase, and α‐amylase—may have contributed indirectly to color development. These enzymes are known to modify the batter matrix and promote the release of reducing sugars and free amino groups, thereby enhancing the availability of reactive substrates involved in Maillard reactions and caramelization processes (Lohinova and Petrusha 2023; Güngör Ertuğral 2021).

**FIGURE 6:**
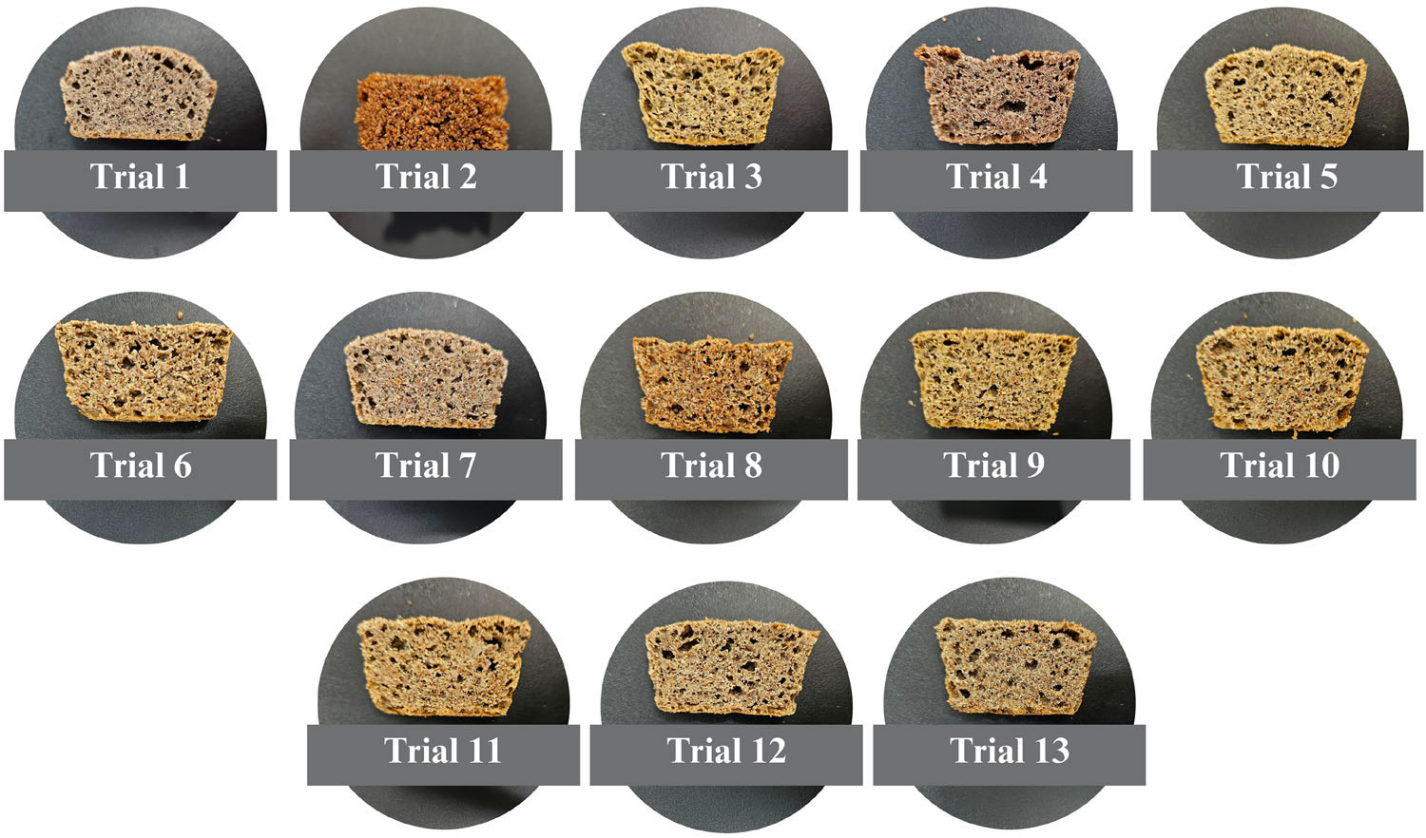
Visual appearance of the top of gluten‐free breads.

**FIGURE 7:**
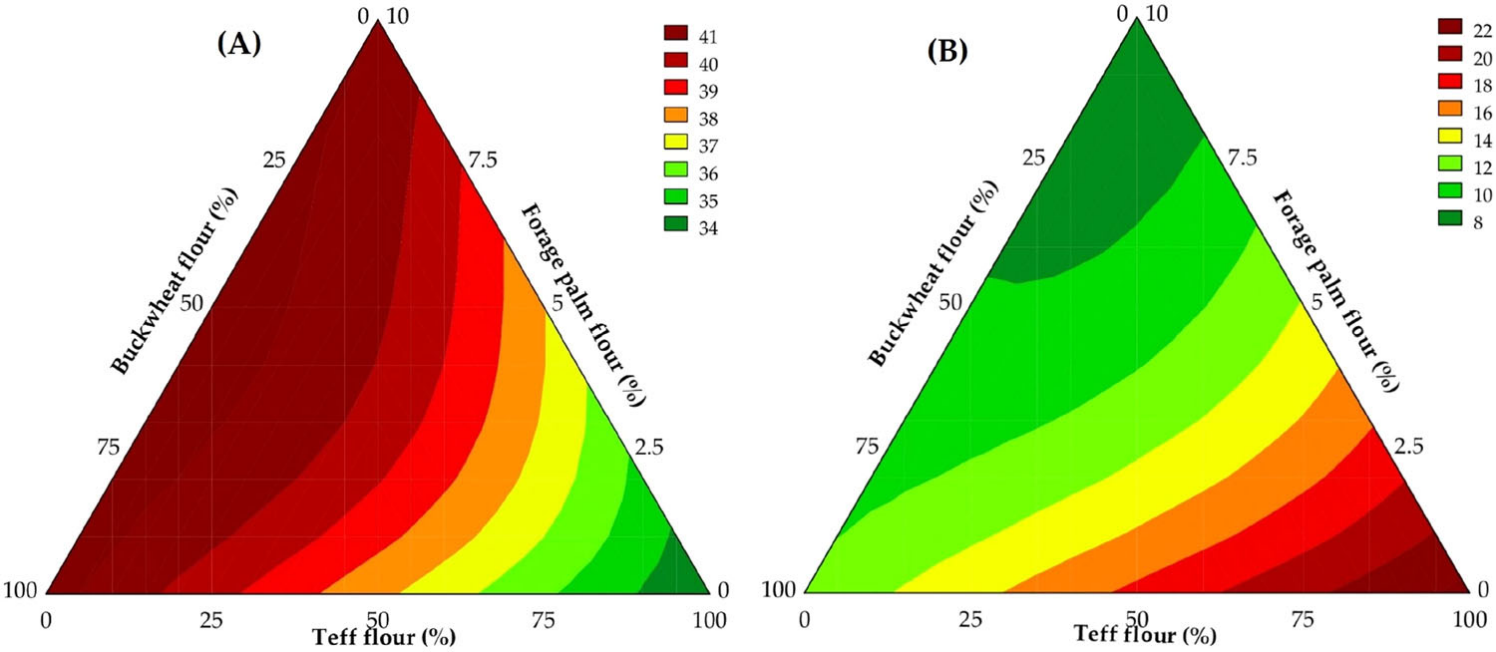
Contour plots illustrate the predicted whiteness index (A) and color index (B) of the gluten‐free bread crumb.

Figure 6 presents a general visual observation of the crumb of the bread obtained from the trials of the experimental design. Whiteness index values of the bread crumb ranged from 33.29 to 41.88, with 96.08% of the variability explained by the predictive mathematical model (Equation 12). A significant model fit was confirmed by ANOVA (*F*_calc_/*F*_tab (3;9;0.05)_ = 19.05, *p* < 0.001). The greatest contributions to the increase in whiteness index (Figure 7A) were attributed to the addition of buckwheat whole flour (*β*_1_ = 41.44, *p* < 0.05) and forage palm whole flour (*β*_3_ = 41.02, *p* < 0.05), whereas teff whole flour exerted a lower influence (*β*_2_ = 33.07, *p* < 0.05). The reduced impact of teff whole flour on whiteness index may be explained by its inherently darker pigmentation, particularly in the brown variety, which is characterized by a high content of luteolin derivatives and phenolic compounds exhibiting strong antioxidant activity (Dueñas et al. 2021; Gebru et al. 2021; Shaltout, 2024).

(12) $$\mathrm{Whiteness}\;\mathrm{index}=41.44x_{1}+33.07x_{2}+41.02x_{3}+33.42x_{1}x_{2}x_{3},$$

where x_1_ = buckwheat whole flour; x_2_ = teff whole flour; and x_3_ = forage palm whole flour.

The intense pigmentation associated with these bioactive compounds directly influences the visual attributes of the formulations by decreasing brightness and, consequently, the whiteness index. Although a reduced whiteness index may be considered a limitation in applications where a lighter appearance is desired, the elevated phenolic and mineral contents of brown teff confer notable nutritional benefits, supporting its inclusion in formulations with an emphasis on functional properties. Moreover, the sensory acceptance of brown teff (particularly in traditional products such as Injera, a staple Ethiopian flatbread) is often linked to its pronounced flavor and distinctive coloration, indicating that the darker hue may be favorably perceived by specific consumer segments (Merchuk‐Ovnat et al. 2020).

The color index values of the breadcrumbs ranged from 6.19 to 22.41, covering color variations from light yellow to the onset of deep orange tones. The predictive mathematical model (Equation 13) accounted for 99.55% of the variability (*F*_calc_/*F*_tab (4;8;0.05)_ = 115.03, *p* < 0.001), indicating high predictability and statistical significance. Teff whole flour (*β*_2_ = 22.58, *p* < 0.001) was identified as the component that most positively influenced the color index, producing crumbs with a more intense coloration, approaching the upper limit of the observed color scale (Figure 7B). This effect may be attributed to the characteristic color of teff grains, which range from light ivory to dark reddish‐brown, depending on the variety. When processed into flour, the color difference of teff becomes less prominent, suggesting that the pigmenting and bioactive compounds, such as flavonoids, are predominantly located in the pericarp of the grain (Gebru et al. 2020; Reta et al. 2022).

(13) $$\begin{array}{ccc} \mathrm{Color}\;\mathrm{index} & = & 10.38x_{1}+22.58x_{2}+6.04x_{3}-8.80x_{2}x_{3}\\ & & -\;38.78x_{1}x_{2}x_{3}, \end{array}$$

where x_1_ = buckwheat whole flour; x_2_ = teff whole flour; and x_3_ = forage palm whole flour.

Buckwheat flour (*β*_1_ = 8.40, *p* < 0.001) and forage palm whole flour (*β*_3_ = 6.14, *p* < 0.001) also contributed to an increase in color index, although the products remained within the light yellow to light orange color range. This modification is likely due to the presence of natural pigments in forage palm, particularly chlorophylls and carotenoids. During the cooking process, chlorophyll, which predominates in plant tissues, tends to degrade, thereby revealing carotenoid accessory pigments associated with yellowish color (Riaz et al. 2021). This degradation process explains the observed warmer tones, such as light yellow and orange, in the breads. In contrast, the negative interaction between teff whole flour and forage palm whole flour (*β*_23_ = −8.80, *p* < 0.001) reduces the color index, suggesting a detrimental synergism between these components in influencing the color parameter.

#### 3.4.2 Specific Volume, Water Activity, and Moisture of Breads

The data obtained for the dependent variables, including the specific volume of whole loaves and the crumb moisture and water activity, are presented in Table 5.

**TABLE 5:** Experimental data regarding specific volume, water activity, and moisture content of gluten‐free breads.

Trial	Specific volume (cm^3^·g^−1^)	Water activity	Moisture content (%)
1	1.96 ± 0.07	0.9684 ± 0.01	37.17 ± 0.03
2	1.85 ± 0.01	0.9558 ± 0.01	38.14 ± 2.08
3	2.47 ± 0.07	0.9774 ± 0.01	46.69 ± 0.21
4	2.12 ± 0.03	0.9575 ± 0.01	46.78 ± 0.22
5	2.01 ± 0.08	0.9592 ± 0.01	41.00 ± 0.24
6	2.04 ± 0.07	0.9454 ± 0.01	37.48 ± 0.32
7	1.99 ± 0.04	0.9536 ± 0.01	36.29 ± 0.20
8	2.16 ± 0.06	0.9529 ± 0.01	36.59 ± 0.28
9	2.08 ± 0.05	0.9642 ± 0.01	43.47 ± 0.23
10	2.12 ± 0.03	0.9614 ± 0.01	38.37 ± 0.09
11	2.12 ± 0.06	0.9489 ± 0.01	37.36 ± 0.13
12	1.90 ± 0.05	0.9558 ± 0.01	37.94 ± 0.15
13	1.97 ± 0.04	0.9541 ± 0.01	36.82 ± 0.26

*Note*: Data are the means of three replicates ± standard deviation.

The specific volume of bread is recognized as a critical indicator of product quality, as it is inversely related to density and reflects the ability of macromolecular interactions and network formation to trap gases during fermentation. Enzymes such as xylanases are often associated with improvements in this parameter, as their hydrolytic action on arabinoxylans reduces dough viscosity, enhances hydration, and strengthens the batter structure (Basit et al. 2025). These effects improve gas retention and contribute to better crumb expansion, while also promoting superior texture and extended shelf life, ultimately leading to increased bread volume. When evaluated alongside density, this parameter provides insight into the ratio of solids to air within the baked matrix. Breads with low specific volume, corresponding to high density, tend to be more compact and chewy, often exhibiting reduced sensory attributes such as crumb softness, flavor, and aroma, whereas breads with higher specific volume are lighter and more aerated (Monteiro et al. 2021). In the present study, the specific volume of the formulations ranged from 1.85 to 2.47 cm^3^·g^−1^. Although 76.53% of the experimental variation was explained by the mathematical model, the *F*‐ratio (*F*_calc_/*F*_tab (6;6;0.05)_ = 0.84) and *p*‐value (*p* = 0.088) indicated that the model lacked predictive capacity, thus precluding the construction of a contour plot. However, the analysis of the regression coefficients revealed a positive effect of the pseudocomponents on the specific volume of the gluten‐free breads. Among the ingredients evaluated, forage palm whole flour exhibited the greatest contribution (*β*_3_ = 2.45, *p* < 0.001), followed by buckwheat whole flour (*β*_1_ = 1.95, *p* < 0.001) and teff whole flour (*β*_2_ = 1.89, *p* < 0.001).

Moisture content ranged from 36.29 to 46.78 g·100 g^−1^. Similarly, no statistically significant effects of the independent variables were observed, as evidenced by an *R* ^2^ value of 75.63% and a *p*‐value of 0.014, confirming that the model did not possess adequate predictive power in this context. Water activity values varied from 0.9454 to 0.9774, as presented in Table 5. No statistically significant influence of the independent variables was detected. The ANOVA yielded an *R* ^2^ value of 67.53% and a *p*‐value of 0.014, indicating insufficient model predictability for this parameter. The higher WAC of the forage palm whole flour (Figure 2) was identified as the main factor responsible, as a pseudocomponent, for the highest observed values of moisture (*β*_3_ = 46.14, *p* < 0.001) and water activity (*β*_3_ = 0.9783, *p* < 0.001). However, the binary interaction between forage palm whole flour and teff whole flour reduced water activity (*β*_23_ = −0.0723, *p* < 0.001), a phenomenon attributed to hydrogen bonding between starchy and non‐starchy polysaccharides and water molecules. In contrast, the ternary interaction among the independent variables decreased the moisture content (*β*_123_ = −168.38, *p* < 0.001) of the gluten‐free breads.

#### 3.4.3 Instrumental Texture of Gluten‐Free Bread Crumb

The texture profile is influenced by diverse physical properties of food structural components, assessed through sensory or instrumental texture analyses. The bread texture profile analysis was performed using a texture analyzer, which subjected the sample to compression in two cycles to simulate chewing (do Nascimento et al. 2024). The results of the instrumental texture analysis of the gluten‐free breads are detailed in Table 6.

**TABLE 6:** Experimental data regarding instrumental texture parameters of gluten‐free breads.

Trial	Firmness (N)	Hardness (N)	Elasticity (%)	Cohesiveness (%)
1	20.82 ± 2.38	23.56 ± 4.20	83.54 ± 1.34	34.97 ± 2.98
2	1.00 ± 0.24	1.72 ± 0.52	13.58 ± 0.94	9.98 ± 1.13
3	4.35 ± 0.28	6.34 ± 0.45	88.46 ± 3.10	56.73 ± 1.82
4	9.85 ± 1.54	12.24 ± 1.52	74.26 ± 2.69	31.20 ± 1.89
5	9.20 ± 1.03	12.57 ± 0.75	86.25 ± 1.97	48.01 ± 2.02
6	6.17 ± 0.80	8.87 ± 1.02	74.67 ± 2.52	39.43 ± 1.79
7	12.56 ± 0.79	15.82 ± 1.75	83.17 ± 1.67	37.78 ± 3.25
8	5.30 ± 0.35	6.81 ± 0.29	65.28 ± 2.56	30.38 ± 2.69
9	6.16 ± 0.57	9.15 ± 0.71	84.97 ± 0.72	50.94 ± 2.31
10	8.33 ± 1.27	11.77 ± 1.92	80.39 ± 2.31	42.11 ± 3.27
11	7.70 ± 1.44	10.70 ± 1.39	81.67 ± 1.81	42.35 ± 2.12
12	9.54 ± 0.73	12.95 ± 1.06	77.85 ± 2.28	38.23 ± 1.65
13	10.86 ± 1.95	13.98 ± 1.73	78.72 ± 2.46	37.41 ± 2.39

Trial	Gumminess (N)	Chewiness (N)	Resilience (%)
1	8.31 ± 2.03	6.95 ± 1.77	17.25 ± 1.38
2	0.18 ± 0.07	0.02 ± 0.01	2.00 ± 0.21
3	3.59 ± 0.27	3.18 ± 0.32	28.54 ± 1.07
4	3.81 ± 0.45	2.83 ± 0.32	12.88 ± 0.73
5	6.03 ± 0.39	5.20 ± 0.36	24.14 ± 1.16
6	3.49 ± 0.41	2.61 ± 0.36	15.61 ± 0.80
7	6.02 ± 1.08	5.01 ± 0.93	18.11 ± 1.72
8	2.07 ± 0.22	1.35 ± 0.17	10.67 ± 1.06
9	4.66 ± 0.39	3.96 ± 0.34	24.50 ± 1.28
10	4.99 ± 1.09	4.00 ± 0.81	18.88 ± 1.52
11	4.52 ± 0.50	3.68 ± 0.38	19.10 ± 0.96
12	4.95 ± 0.41	3.85 ± 0.33	16.84 ± 0.95
13	5.22 ± 0.64	4.11 ± 0.52	16.54 ± 1.25

*Note*: Data are the means of three replicates ± standard deviation.

**FIGURE 8:**
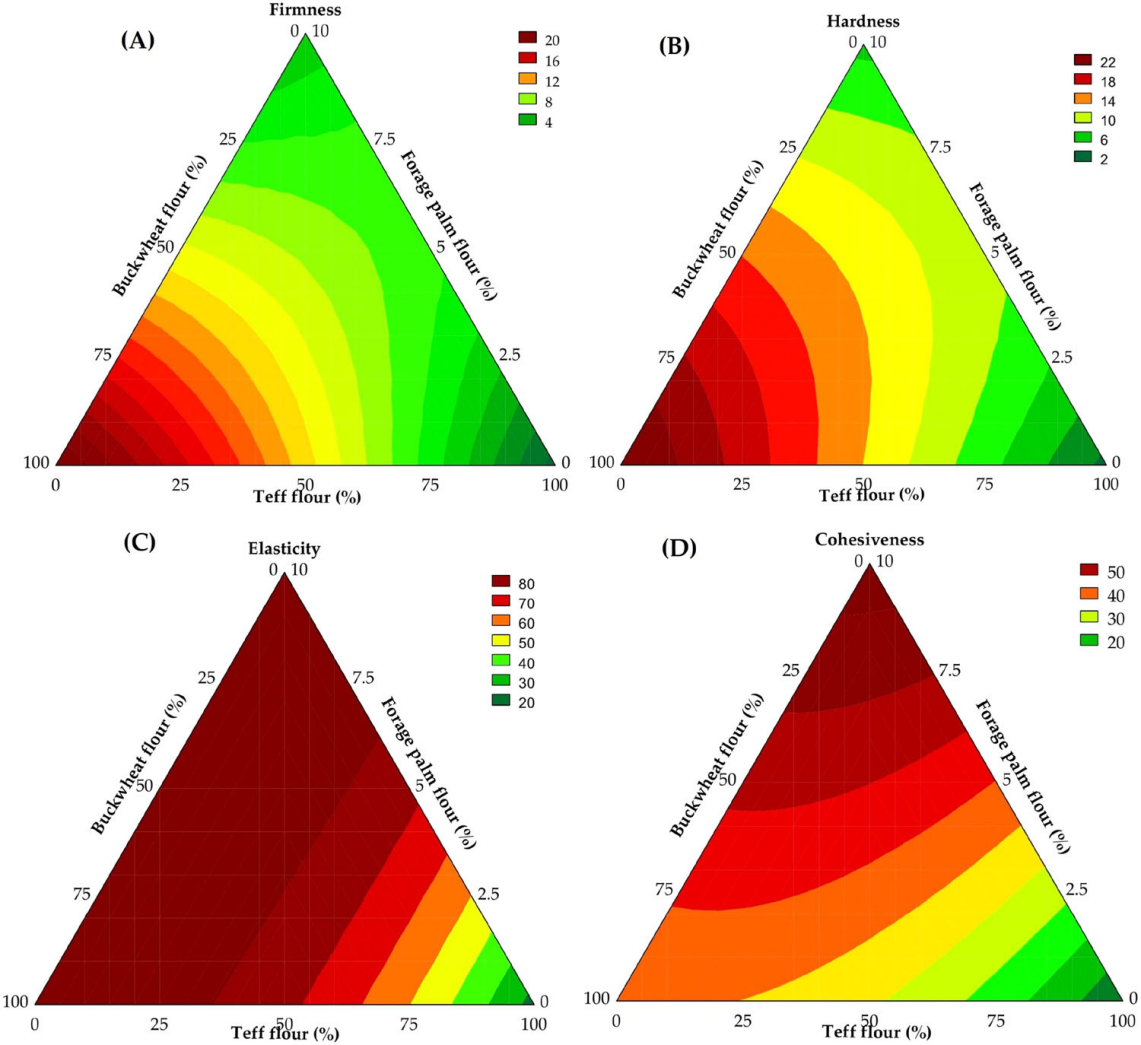
Contour plots illustrate the predicted firmness (A), hardness (B), elasticity (C), and cohesiveness (D) of the gluten‐free bread crumb.

Firmness and hardness of the breads are defined as the force required to deform or rupture the product's structure (Monteiro et al. 2021) and exhibited considerable variation among the formulations, indicating the significant influence of compositional differences on the resulting texture. Firmness ranged from 1.00 to 20.82 N, with the mathematical model (Equation 14) demonstrating a high explanatory capacity (*R* ^2^ = 96.88; *F*_calc_/*F*_tab _= 16.20; *p* < 0.001). Hardness values varied from 1.72 to 23.56 N, with 96.06% of the variability accounted for by the corresponding mathematical model presented in Equation (15) (*F*_calc_/*F*_tab _= 18.97; *p* < 0.001). As illustrated in Figure 8A (for firmness) and Figure 8B (for hardness), the incorporation of buckwheat whole flour significantly increased firmness (*β*_1_ = 20.29, *p* < 0.001) and hardness (*β*_1_ = 22.51, *p* < 0.001), an effect likely attributable to its elevated protein and starch contents, which resulted in a denser and more resistant structural matrix (Zhu 2021), in agreement with textural behavior observed in batter systems. Furthermore, the addition of TGase may have contributed to slight increases in firmness by promoting additional protein crosslinking, while carboxymethyl cellulose may have aided in water retention and crumb stabilization. However, these effects were limited, indicating that the intrinsic composition of the flours played the predominant role in determining the texture of the breads.

(14) $$\begin{array}{ccc} \mathrm{Firmness}\left(N\right) & = & 20.29x_{1}+0.57x_{2}+4.23x_{3}-12.20x_{1}x_{3}\\ & & +\;15.51x_{2}x_{3}, \end{array}$$

where x_1_ = buckwheat whole flour; x_2_ = teff whole flour; and x_3_ = forage palm whole flour.

(15) $$\mathrm{Hardness}\left(N\right)=22.51x_{1}+1.45x_{2}+5.42x_{3}+20.32x_{2}x_{3},$$

where x_1_ = buckwheat whole flour; x_2_ = teff whole flour; and x_3_ = forage palm whole flour.

On the other hand, a favorable behavior was observed through the combination of buckwheat and forage palm whole flours concerning firmness (*β*_13_ = −12.20, *p* = 0.023) and by the contribution of forage palm whole flour to hardness (*β*_3_ = 5.42, *p* = 0.001). Presumably, their high water retention capacity and mucilage content of these flours contribute to a more hydrated and less rigid internal matrix. From a technological standpoint, such an effect is particularly advantageous in gluten‐free formulations, which are often characterized by overly dense and hardened textures that negatively impact sensory acceptance (Šmídová and Rysová 2022). In this context, forage palm flour functions as an alternative structuring agent, promoting softness in the final product. Its mucilaginous compounds, known for their water solubility and gelling and thickening properties, enhance moisture retention and contribute to batter plasticity, thus enabling a lighter and more palatable texture.

Elasticity, defined as the crumb's ability to return to its original shape following the initial compression cycle (de Oliveira Teotônio et al. 2021), ranged from 13.58% to 88.46%, with 98.00% of the variance explained by the predictive model (Equation 16) and a *F*_calc_/*F*_tab (4;8;0.05)_ ratio of 25.54 (*p* < 0.001), enabling the generation of a contour plot (Figure 8C). The addition of the whole flours significantly influenced bread elasticity. Buckwheat whole flour (*β*_1_ = 82.19, *p* < 0.001) and forage palm whole flour (*β*_3_ = 87.12, *p* < 0.001) were the most influential factors, likely due to their elevated water‐holding capacities, which facilitate CO_2_ retention during leavening and promote structural recovery post‐compression. Teff whole flour also contributed positively (*β*_2_ = 16.26, *p* = 0.001), albeit to a lesser extent. The binary interactions among the flours played a critical role. The combined use of buckwheat and teff whole flours (*β*_12_ = 93.40, *p* < 0.001) significantly enhanced elasticity, suggesting a synergistic interaction. Similarly, the interaction between teff and forage palm whole flours (*β*_23_ = 85.21, *p* < 0.001) also increased elasticity, albeit to a slightly lesser degree.

(16) $$\begin{array}{ccc} \mathrm{Elasticity}\left(\%\right) & = & 82.19x_{1}+16.26x_{2}+87.12x_{3}+93.40x_{1}x_{2}\\ & & +\;85.21x_{2}x_{3}, \end{array}$$

where x_1_ = buckwheat whole flour; x_2_ = teff whole flour; and x_3_ = forage palm whole flour.

Breads exhibiting low cohesiveness generally tend to crumble easily, an undesirable characteristic (Pontes et al. 2020). Cohesiveness values ranged from 9.98% to 56.73%, with 97.96% of the data variation explained by the mathematical model (Equation 17) (*F*_calc_/*F*_tab (4;8;0.05)_ = 24.98, *p* < 0.001). The highest cohesiveness values were observed in breads formulated with forage palm whole flour (*β*_3_ = 57.72, *p* < 0.001), followed by those with buckwheat whole flour (*β*_1_ = 35.10, *p* < 0.001). Teff whole flour also contributed positively, albeit to a lesser extent (*β*_2_ = 10.57, *p* = 0.001). The interaction between buckwheat and teff whole flours (*β*_12_ = 31.71, *p* = 0.006) further enhanced cohesiveness, suggesting that their combination is more effective than their individual use. Similarly, the interaction between teff and forage palm whole flours (*β*_23_ = 22.84, *p* = 0.027) also improved cohesiveness, although to a slightly lesser extent, as can be observed in Figure 8D.

(17) $$\begin{array}{ccc} \mathrm{Cohesiveness}\left(\%\right) & = & 35.10x_{1}+10.57x_{2}+57.72x_{3}+31.71x_{1}x_{2}\\ & & +\;22.84x_{2}x_{3}, \end{array}$$

where x_1_ = buckwheat whole flour; x_2_ = teff whole flour; and x_3_ = forage palm whole flour.

**FIGURE 9:**
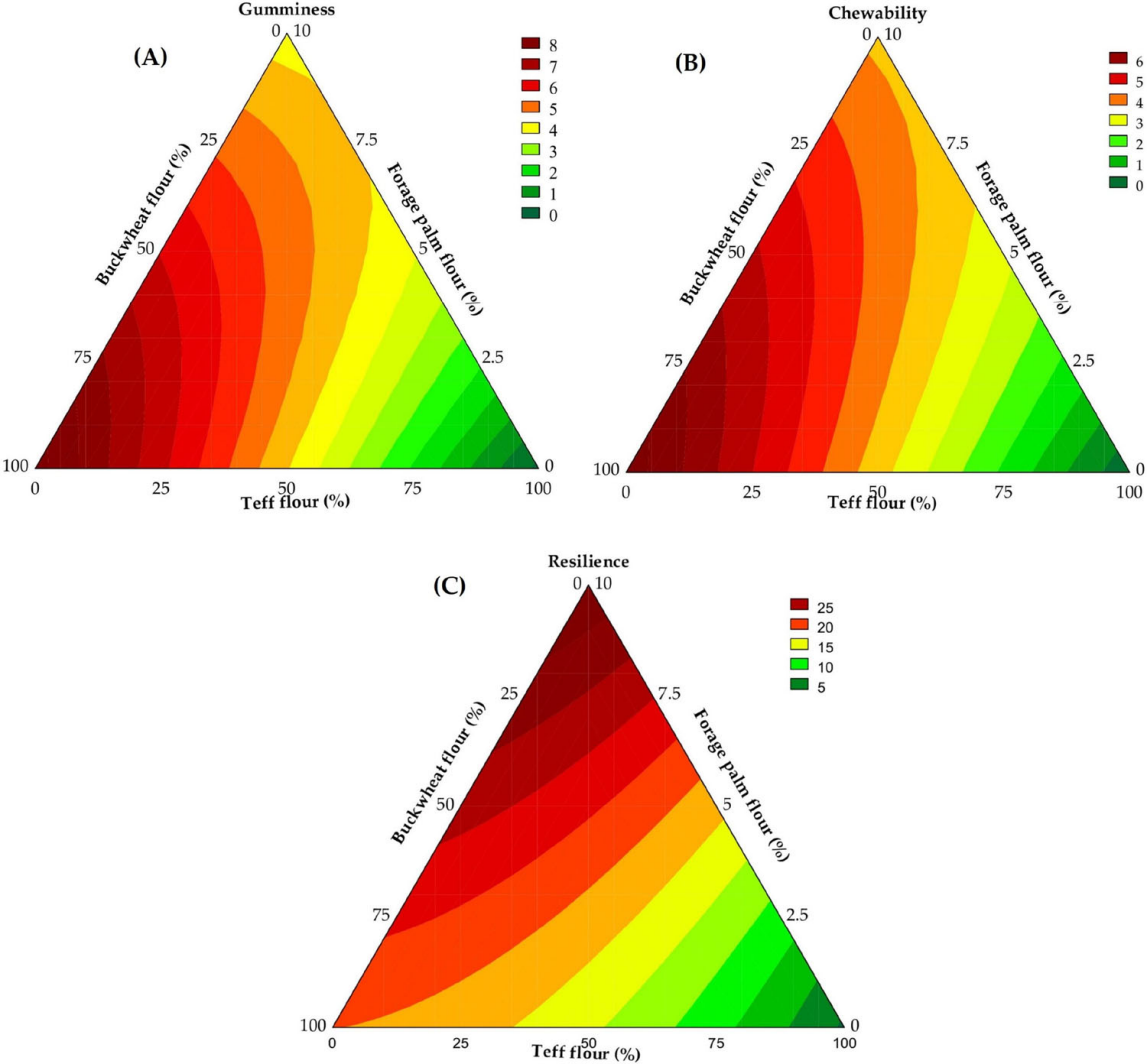
Contour plots illustrate the predicted gumminess (A), chewiness (B), and resilience (C) of the gluten‐free bread crumb.

Gumminess is defined as the force required to masticate the sample until it is ready for swallowing, and chewiness refers to the energy necessary for disintegration before swallowing, which are textural attributes for which lower values are preferred in soft crumb products (do Nascimento et al. 2024). Gumminess ranged from 0.18 to 8.31 N, with 97.03% of the variability explained by the predictive model (Equation 18) and a *F*_calc_/*F*_tab (3;9;0.05)_ ratio of 34.06 (*p* < 0.001). The highest positive effect on gumminess was attributed to buckwheat whole flour (*β*_1_ = 8.22, *p* < 0.001), followed by forage palm whole flour (*β*_3_ = 3.73, *p* < 0.001). A significant increase in gumminess was also observed for the interaction between teff and forage palm whole flours (*β*_23_ = 7.08, *p* = 0.001). According to Figure 9A, lower gumminess values were obtained with lower contents of buckwheat and forage palm whole flours and higher concentrations of teff whole flour.

(18) $$\mathrm{Gumminess}\left(N\right)=8.22x_{1}-0.15x_{2}+3.73x_{3}+7.08x_{2}x_{3}$$

where x_1_ = buckwheat whole flour; x_2_ = teff whole flour; and x_3_ = forage palm whole flour.

Chewiness values varied from 0.02 to 6.95 N, with 97.36% of the results explained by the predictive mathematical model (*F*_calc_/*F*_tab (3;9;0.05)_ = 28.64, *p* < 0.001) presented in Equation (19). The highest chewiness values were associated with the inclusion of buckwheat whole flour (*β*_1_ = 6.82, *p* < 0.001), followed by the interaction between teff and forage palm whole flours (*β*_23_ = 4.59, *p* = 0.008) and forage palm whole flour as a pseudocomponent (*β*_3_ = 3.37, *p* < 0.001). Lower chewiness values were obtained with higher levels of teff whole flour and lower concentrations of buckwheat and forage palm whole flours in the formulation of the gluten‐free breads (Figure 9B).

(19) $$\mathrm{Chewiness}\left(N\right)=6.82x_{1}-0.40x_{2}+3.37x_{3}+4.59x_{2}x_{3}$$

where x_1_ = buckwheat whole flour; x_2_ = teff whole flour; and x_3_ = forage palm whole flour.

Resilience represents the sample's capability to recover its original shape following deformation and directly reflects its elastic properties (Wang et al. 2024). The resilience of the samples ranged from 2.00% to 28.54%, with 98.43% of the variability explained by the mathematical model (Equation 20), which was considered predictive due to presenting a *F*_calc_/*F*_tab (3;9;0.05)_ ratio of 48.85 and a significance level lower than 0.001. The greatest increases in resilience were associated with the addition of buckwheat whole flour (*β*_1_ = 17.57, *p* < 0.001) and forage palm whole flour (*β*_3_ = 29.54, *p* < 0.001), while teff whole flour exhibited a modest contribution (*β*_2_ = 2.08, *p* = 0.033). Moreover, the interaction between buckwheat and teff whole flours (*β*_12_ = 12.62, *p* = 0.012) enhanced resilience. As observed in Figure 9C, similar behaviors were found for resilience, gumminess, and chewiness concerning the presence of buckwheat, teff, and forage palm whole flours.

(20) $$\mathrm{Resilience}\left(\%\right)=17.57x_{1}+2.08x_{2}+29.54x_{3}+12.62x_{1}x_{2}$$

where x_1_ = buckwheat whole flour; x_2_ = teff whole flour; and x_3_ = forage palm whole flour.

In summary, buckwheat flour was identified as the primary contributor to the increased gumminess and chewiness of the breads, a trend consistent with its influence on batter firmness and hardness. This behavior is likely related to its high WAC, which contributes to developing a denser, less extensible structure, affecting the final textural quality of the gluten‐free breads.

### 3.5 Numerical Optimization and Validation of Mathematical Models

The optimization was carried out based on the physicochemical and technological characteristics of the gluten‐free batters and breads. For the dependent variables that were statistically significant (*R* ^2^ > 0.80; *F*_calc_/*F*_tab_ > 1.0; and *p*‐value < 0.05), importance was assigned using a scale from 1 to 5, where 1 represented the lowest importance and 5 the highest. The dependent variables that demonstrated statistical significance were optimized to minimize batter adhesiveness and viscosity index, as well as crumb firmness, hardness, gumminess, and chewiness. Conversely, the responses associated with batter firmness and consistency, along with crumb elasticity and resilience, were maximized. Meanwhile, the values of batter pH, crumb cohesiveness, and colorimetric parameters were maintained within range.

The independent variables were maintained within the specified range, and the optimized formulation was obtained using 91.4% buckwheat whole flour and 8.6% forage palm whole flour, resulting in a desirability index of 65.50%. This formulation was designated as the optimized point without teff whole flour (OP − TWF) (Table 7).

**TABLE 7:** Numerical optimization, experimental and predicted values, and relative deviation for the validation of the mathematical models for the optimal point without teff whole flour.

Parameter	Goal	Importance	Solution	
			Coded values	Real values
Independent variables				
Buckwheat whole flour	In range	3	0.14	91.4%
Teff whole flour	In range	3	0	0
Forage palm whole flour	In range	3	0.86	8.6%

Parameter	Goal	Importance	Solution	Experimental value	Relative deviation (%)
Dependent variables					
pH | batter	In range	3	4.89	5.01 ± 0.03	2.49
Firmness (N) | batter	Maximize	5	0.49	0.62 ± 0.01	26.72
Consistency (N·s)	Maximize	5	12.43	16.62 ± 0.22	33.73
Adhesiveness (N)	Minimize	5	−0.29	−0.36 ± 0.01	23.50
Viscosity index (N·s)	Minimize	5	−7.78	−10.04 ± 1.46	28.98
Whiteness index	In range	3	41.08	38.74 ± 0.30	−5.69
Color index	In range	3	6.65	6.57 ± 0.23	−1.30
Firmness (N) | crumb	Minimize	5	5.01	7.95 ± 0.66	58.59
Hardness (N)	Minimize	5	7.83	10.73 ± 0.65	36.99
Elasticity (%)	Maximize	5	86.42	85.07 ± 0.34	−1.57
Cohesiveness (%)	In range	3	56.73	50.77 ± 0.11	−13.41
Gumminess (N)	Minimize	5	4.36	5.26 ± 0.14	20.61
Chewiness (N)	Minimize	5	3.86	4.48 ± 0.10	16.09
Resilience (%)	Maximize	5	27.85	24.27 ± 0.77	12.86
Desirability = 65.50%					

Considering the favorable effects observed on batter and gluten‐free bread properties, as well as the recognized nutritional value of teff flour, attributable to its elevated levels of proteins, dietary fiber, and essential minerals such as iron and calcium, a subsequent numerical optimization was conducted. In this new approach, 10% teff whole flour was considered in the formulation, while the proportions of buckwheat and forage palm whole flours were kept in range (Table 8). Under these conditions, the optimized formulation consisted of 81.8% buckwheat whole flour, 10% teff whole flour, and 8.2% forage palm whole flour, with a desirability index of 63.80%. This formulation was identified as the optimized point with teff whole flour (OP + TWF).

**TABLE 8:** Numerical optimization, experimental and predicted values, and relative deviation for the validation of the mathematical models for the optimal point with teff whole flour.

Parameter	Goal	Importance	Solution	
			Coded values	Real values
Independent variables				
Buckwheat whole flour	In range	3	0.08	81.8%
Teff whole flour	Is target (0.10)	5	0.10	10.0%
Forage palm whole flour	In range	3	0.82	8.2%

Parameter	Goal	Importance	Solution	Experimental value	Relative deviation (%)
Dependent variables					
pH | batter	In range	3	4.85	4.96 ± 0.02	2.27
Firmness (N) | batter	Maximize	5	0.42	0.36 ± 0.01	−14.75
Consistency (N·s)	Maximize	5	10.70	9.15 ± 0.12	−14.50
Adhesiveness (N)	Minimize	5	−0.25	−0.21 ± 0.01	−15.09
Viscosity index (N·s)	Minimize	5	−6.77	−4.08 ± 0.10	−39.83
Whiteness index	In range	3	40.49	37.94 ± 0.75	−6.28
Color index	In range	3	7.07	7.15 ± 0.43	1.11
Firmness (N) | crumb	Minimize	5	5.64	5.52 ± 0.22	−2.00
Hardness (N)	Minimize	5	8.11	7.95 ± 0.30	−2.07
Elasticity (%)	Maximize	5	87.36	84.83 ± 0.46	−2.89
Cohesiveness (%)	In range	3	53.45	50.77 ± 0.11	−5.00
Gumminess (N)	Minimize	5	4.30	4.03 ± 0.14	−6.24
Chewiness (N)	Minimize	5	3.66	3.42 ± 0.10	−6.59
Resilience (%)	Maximize	5	25.89	24.49 ± 0.28	−5.43
Desirability = 63.80%					

### 3.6 Centesimal Analysis and Caloric Value of Optimized Gluten‐Free Breads

The breads produced from the optimized formulations were characterized based on their proximate composition, which included the determination of moisture, protein, lipid, ash, dietary fiber, and digestible carbohydrate contents (starch and sugars). Additionally, the total caloric value per serving (50 g) was calculated. The results of the proximate composition and caloric content corresponding to the optimized formulations are presented in Table 9.

**TABLE 9:** Proximate composition (g·100 g^−1^) and total caloric value (calories per 50 g of serving) of gluten‐free breads.

Formulation	OP − TWF	OP + TWF	*p*‐value
Moisture	41.17 ± 0.21	42.03 ± 0.27	0.257
Proteins	11.81 ± 0.61	11.66 ± 0.07	0.738
Lipids	3.61 ± 0.07	3.28 ± 0.49	0.394
Ashes	2.89 ± 0.01	2.80 ± 0.02	0.001
Total dietary fibers	11.44 ± 1.07	8.56 ± 0.42	0.010
Digestible carbohydrates	29.08	31.67	—
Total caloric value	98.03	101.42	—

*Note*: Data are the means of 10 replicates ± standard deviation.

Abbreviations: OP − TWF, optimal point without teff whole flour; OP + TWF, optimal point with teff whole flour.

No significant difference was observed in the moisture content of the optimized formulations, indicating that the inclusion of teff flour did not influence this parameter. The value obtained (41.17%) was close to that reported by Lima et al. (2021), who found a moisture content of 40.37% in a similar gluten‐free formulation. This elevated moisture level is commonly observed in gluten‐free breads and is related to the need for greater water incorporation during processing, aiming to improve softness, volume, and freshness. Such characteristics are associated with the distinct water absorption properties of alternative flours compared to traditional wheat flour.

The protein contents of the OP − TWF and OP + TWF samples were 11.81% and 11.66%, respectively, with no statistically significant difference, suggesting that the addition of teff flour did not affect the protein content of the formulations. The elevated protein levels, characteristic of protein‐enriched breads, may be attributed to the presence of albumin in the formulations. These values were notably higher than those reported by Tsatsaragkou et al. (2014), who incorporated 12.5 g of carob flour, 15 g of resistant starch, and 10 g of carob protein into gluten‐free breads, resulting in a final protein content of 8.37%. Higher values were also reported by Brites et al. (2022) in gluten‐free breads containing buckwheat flour, with protein levels of 10.50%, 12.71%, and 13.05% for formulations F1 (100% buckwheat flour), F2 (70% buckwheat flour and 30% wheat flour), and F3 (55% buckwheat flour and 45% wheat flour), respectively.

According to the data presented in Table 9, no significant difference was observed in the lipid content between OP − TWF and OP + TWF, as determined by the Student's *t*‐test, indicating that the inclusion of teff flour did not influence this parameter. This result is consistent with the equal amounts of palm fat incorporated into both formulations. Palm fat is known for its balanced fatty acid profile, with approximately equal proportions of saturated and unsaturated fatty acids, which supports its use as a substitute for hydrogenated fat in various food applications (Sulaiman et al. 2022).

The ash content of the optimized formulations was 2.89% for OP − TWF and 2.80% for OP + TWF, with a statistically significant difference (*p* = 0.001). However, from a nutritional perspective, this difference is considered negligible, suggesting that the incorporation of teff flour had no meaningful impact on the total mineral content. Nonetheless, teff flour is recognized as a source of essential minerals, contributing macroelements such as phosphorus (P), potassium (K), calcium (Ca), magnesium (Mg), and sodium (Na), as well as microelements including iron (Fe), zinc (Zn), manganese (Mn), copper (Cu), and molybdenum (Mo), in higher concentrations compared to cereals such as corn and wheat (Gebru et al. 2020).

A statistically significant difference was observed in the dietary fiber content of the optimized formulations (*p* = 0.010), with OP − TWF exhibiting a higher fiber content. The inclusion of teff flour resulted in a reduction in total dietary fiber, indicating that the higher fiber content in OP − TWF was primarily due to the greater proportion of buckwheat flour. Buckwheat is recognized as a nutrient‐dense pseudocereal, rich in resistant starch, digestible carbohydrates, proteins, vitamins, minerals, and particularly dietary fibers. Its high fiber content plays an important role in preventing conditions such as constipation and obesity, highlighting its nutritional relevance (Ruan et al. 2022).

The digestible carbohydrate content of the optimized formulations was similar, with OP + TWF presenting a slightly higher value (31.67%) compared to OP − TWF (29.08%). This minor variation suggests that both formulations provide comparable amounts of digestible carbohydrates, with OP + TWF offering a marginally higher intake. The observed difference may be attributed to the differing proportions of ingredients used in the formulations, particularly the ratio of buckwheat to teff flour, which directly influences the digestible carbohydrate content.

### 3.7 Total Soluble Phenolic Compounds and Antioxidant Capacity of Optimized Points

The total soluble phenolic compound contents were determined to be 195.17 ± 10.83 mg GAE·100 g^−1^ for OP − TWF and 95.18 ± 8.35 mg GAE·100 g^−1^ for OP + TWF, with a statistically significant difference (*p* < 0.05). Regarding these compounds, the OP − TWF formulation—composed predominantly of buckwheat and forage palm whole flours—exhibited a significantly higher concentration when compared to OP + TWF. This result may be attributed to the absence of teff flour in the formulation, which led to a proportional increase in the amounts of buckwheat and forage cactus flours, both recognized for their substantial contributions to the phenolic compound content. This behavior is consistent with the findings of Brites et al. (2022), who demonstrated that the incorporation of buckwheat flour significantly enhanced the levels of phenolic compounds such as rutin and quercetin.

The antioxidant capacities of the samples, quantified by the ORAC method, were 75.23 ± 0.33 µmol TE·100 g^−1^ for OP − TWF and 72.73 ± 1.07 µmol TE·100 g^−1^ for OP + TWF, indicating that the OP − TWF formulation exhibited superior performance in terms of bioactive compound content and antioxidant activity. Similarly, Eren and Akkaya (2024) reported that the addition of buckwheat flour led to a substantial increase in phenolic compound content and antioxidant activity in bread formulations. In their study, bread containing 20% buckwheat flour showed 116.2 mg GAE·100 g^−1^ and an antioxidant capacity of 27.2 mmol TE·100 g^−1^, which were significantly higher than those observed in the control bread (43.6 mg GAE·100 g^−1^ and 6.86 mmol TE·100 g^−1^, respectively). Despite these increases, the values reported in the present study were considerably higher.

Concerning antioxidant capacity as determined by the ORAC method, it was noted that although the absolute difference between the two breads was smaller than that observed for total phenolic compound content, the antioxidant capacities of both formulations remained substantially higher than those reported by Eren and Akkaya (2024). These findings suggest that the elevated concentration of bioactive compounds, particularly those derived from buckwheat and forage cactus flours, played a critical role in enhancing the antioxidant potential of the breads. Furthermore, the results indicate that the antioxidant properties were more effectively preserved in the present study when compared to values commonly reported in the literature.

### 3.8 Sensorial Analysis

**FIGURE 10:**
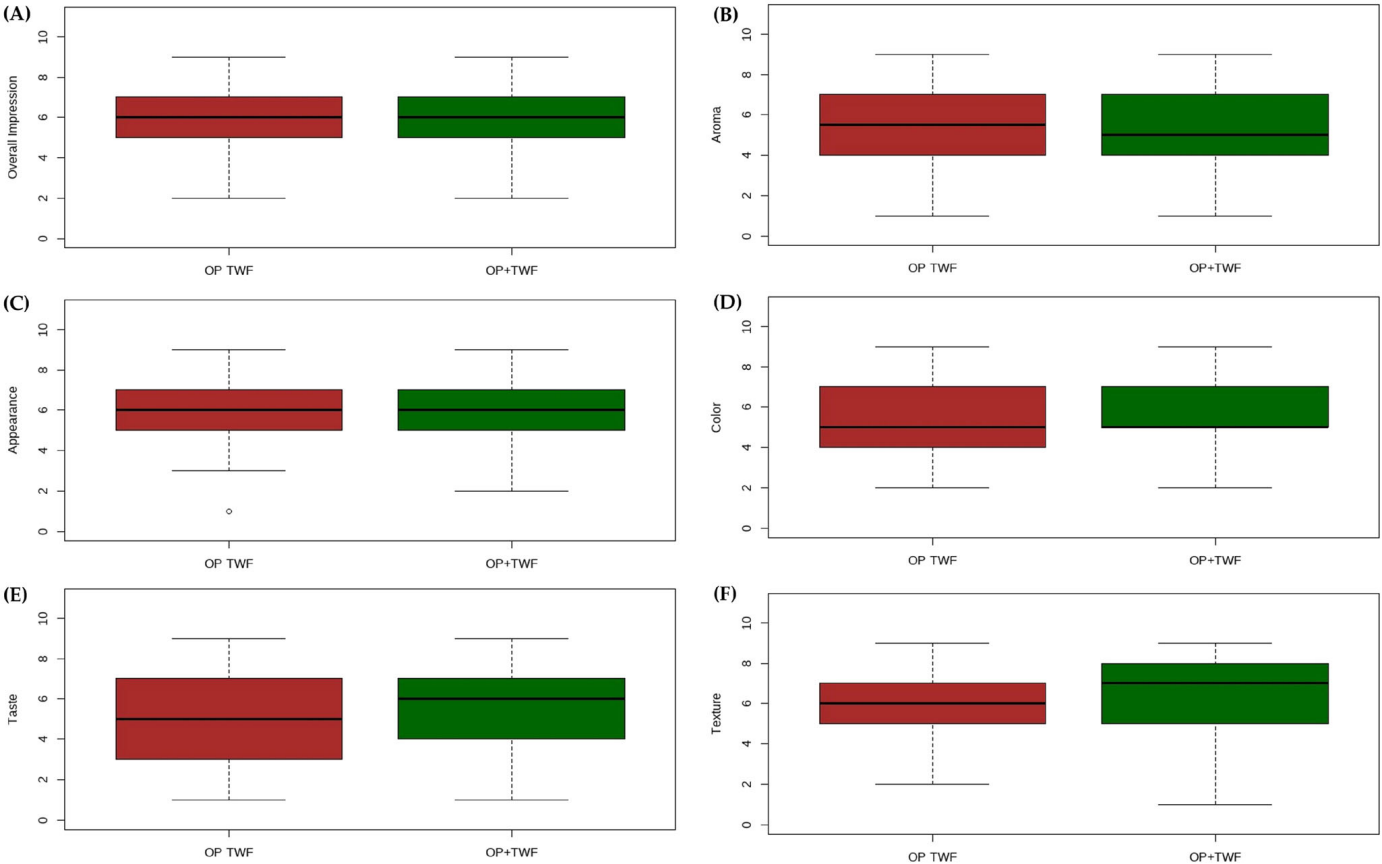
Medians obtained from the acceptance test of optimized points considering overall impression (A), aroma (B), appearance (C), color (D), taste (E), and texture (F). OP − TWF, optimal point without teff whole flour; OP + TWF, optimal point with teff whole flour.

Sensory evaluations were conducted on the samples corresponding to the optimized points of the gluten‐free breads (Table 10). Based on the Focus Group sessions, the following descriptors were identified: acidic, brown, greenish, whole‐grain bread flavor, crumbly, plant‐like odor, soft, plant‐like taste, compact, whole‐grain appearance, aftertaste, grain odor, melts in the mouth, sandy, speckled, dry, fibrous consistency, porous, and fibrous appearance. The results of the Acceptance Test, conducted using the 9‐point Hedonic Scale (1 = *Dislike extremely*; 2 = *Dislike very much*; 3 = *Dislike moderately*; 4 = *Dislike slightly*; 5 = *Neither like nor dislike*; 6 = *Like slightly*; 7 = *Like moderately*; 8 = *Like very much*; 9 = *Like extremely*), are presented in Figure 10.

**TABLE 10:** Formulations of gluten‐free breads from sensorially evaluated samples, expressed in grams.

Ingredients/technological aids	OP − TWF	OP + TWF
Buckwheat whole flour	376.28	336.75
Teff whole flour	0	41.17
Forage palm whole flour	35.4	33.76
Egg albumin	20.58	20.58
Sucrose	16.47	16.47
Instant dry yeast	8.23	8.23
Palm fat	16.47	16.47
Iodized kitchen salt	7.41	7.41
Carboxymethylcellulose	6.18	6.18
Phospholipase	0.0062	0.0062
Transglutaminase	0.0412	0.0412
α‐Amylase	0.0041	0.0041
Xylanase	0.0124	0.0124
Potable water	435.2 (CAA = 2.92)	460.5 (CAA = 3.09)

*Note*: The amount of water was adjusted considering the water absorption capacity (WAC) of the optimized blends, which was 2.92 g/g for OP − TWF and 3.09 g/g for OP + TWF.

Abbreviations: OP − TWF, optimal point without teff whole flour; OP + TWF, optimal point with teff whole flour.

Both formulations exhibited similar levels of acceptance concerning overall impression, aroma, appearance, and color. However, significant differences were observed in texture and flavor. OP + TWF received a median score of 7 (moderately liked) for texture, compared to a median score of 6 (slightly liked) for OP − TWF, indicating a higher level of acceptance for OP + TWF. Similarly, in terms of flavor, OP + TWF received a median score of 5 (neither liked nor disliked), while OP − TWF received a median score of 6 (slightly liked). Overall, while the formulations were equivalent in several sensory attributes, OP + TWF was preferred due to its superior texture and flavor.

**FIGURE 11:**
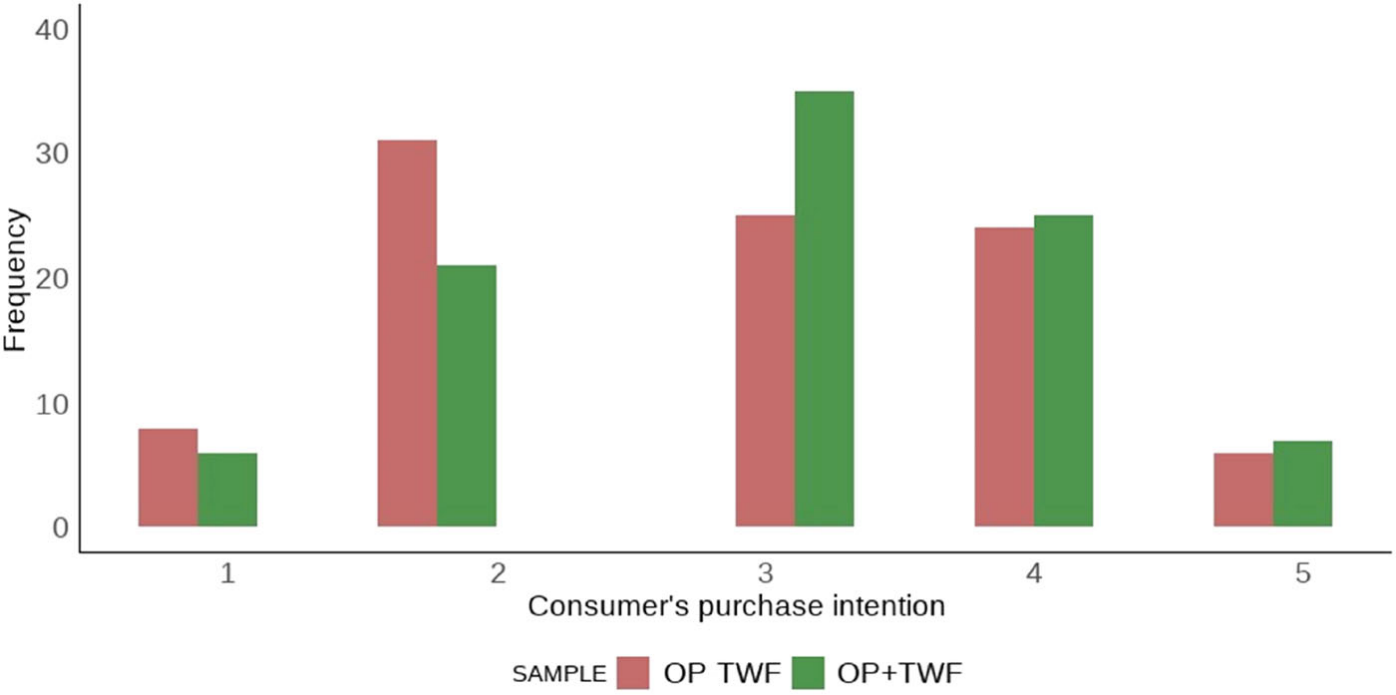
Result of the purchase intention test of optimized points. OP − TWF, optimal point without teff whole flour; OP + TWF, optimal point with teff whole flour.

Regarding purchase intention (Figure 11), many panelists expressed uncertainty about purchasing either formulation, with the OP + TWF sample receiving more uncertainty than the OP − TWF sample. This could be attributed to the fact that gluten‐free products are not part of the participants’ regular diets, as most do not have celiac disease or any restrictions on gluten consumption. However, a portion of the panelists indicated a likelihood to purchase the product, with similar purchase intention values observed for both OP + TWF (26.60%) and OP − TWF (25.53%) samples.

**FIGURE 12:**
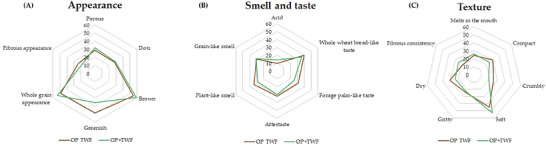
Results of the Check‐All‐That‐Apply test for the optimized points for appearance (A), smell and taste (B), and texture (C). OP − TWF, optimal point without teff whole flour; OP + TWF, optimal point with teff whole flour.

The results of the CATA test descriptors are presented in Figure 12. Concerning odor and taste, the samples exhibited similar behavior for the term “grain odor,” which can be explained using the respective flours. The descriptors “whole grain bread taste,” “aftertaste,” “plant taste,” and “plant odor” were more frequently mentioned for OP + TWF. Despite the slightly lower amount of palm flour in OP + TWF, the higher frequency of reports related to the vegetable descriptors was observed experimentally, indicating that the incorporation of teff flour contributed to these sensory perceptions.

The “whole grain appearance” of the breads was identified as a determining factor for product acceptance. The predominance of the brown appearance, as also mentioned, contributed significantly to this positive perception. Conversely, a greenish appearance was more frequently reported for the OP − TWF sample, which may be attributed to the natural pigments present in the palm flour. These pigments, known for their greenish hue, were likely perceptible to the evaluators during the sensory analysis.

The texture of the breads was described using descriptors such as “crumbly,” “compact,” “melt‐in‐the‐mouth,” “fibrous consistency,” “dry,” “sandy,” and “soft,” with “soft” being the most prominent, particularly for the OP + TWF formulation. This improvement in softness was experimentally observed in the texture profile analysis, which demonstrated that the inclusion of teff flour resulted in less firmness, indicating a less rigid matrix, and greater cohesiveness, reflecting a more cohesive internal structure capable of maintaining its integrity during chewing. These instrumental data corroborate the sensory evaluation, confirming that teff contributed to a smoother, more uniform, and more pleasant textural experience, especially in the OP + TWF formulation. The other ingredients also influenced the perception of attributes such as crumbliness and fibrous consistency, resulting in a complex and balanced texture, in which each component contributes to the final characteristics of the bread.

## 4 Conclusion

The results indicated that forage palm whole flour exhibited superior performance regarding batter and gluten‐free bread characteristics, followed by buckwheat and teff whole flours. Notable improvements were observed in batter firmness and adhesiveness, as well as crumb firmness and elasticity. The formulation composed of 81.8% buckwheat, 10% teff, and 8.2% forage palm whole flour showed the best performance in both mathematical model validation and sensory analysis, receiving the highest scores for appearance, texture, overall acceptance, and purchase intent. Sensory descriptors suggested that teff whole flour contributed to a softer texture, greater flavor complexity, and enhanced “melts in the mouth” perception, while reducing undesirable attributes such as “gritty” and “crumbly.” Visual characteristics were positively affected, with the OP + TWF formulation exhibiting a more pronounced whole grain‐like appearance, likely due to its deeper brown coloration, whereas the OP − TWF formulation appeared more greenish due to the natural pigments in the forage palm whole flour. Overall, the inclusion of 10% teff whole flour improved both the sensory and nutritional quality of gluten‐free bread, demonstrating superiority over the formulation without teff.

## Nomenclature

AACCI: American Association of Cereal Chemists International
AAPH: 2,2′‐azobis(2‐methylpropionamidine) dihydrochloride
ANOVA: analysis of variance
CATA: Check All That Apply
GAE: gallic acid equivalents
OP−TWF: optimal point without teff whole flour
OP+TWF: optimal point with teff whole flour
ORAC: oxygen radical absorbance capacity
TE: Trolox equivalent
WAC: water absorption capacity

## Author Contributions

**Mateus Alves Araújo**: methodology, software, formal analysis, visualization, investigation, writing – original draft. **Nathalia de Andrade Neves**: methodology, software, validation, data curation, formal analysis, investigation, visualization. **Irene Andressa**: investigation, data curation, writing – original draft, visualization. **Tatiana Nunes Amaral**: methodology, software, validation, formal analysis, investigation, resources, data curation, writing – original draft, visualization, project administration. **Marcio Schmiele**: conceptualization, validation, resources, data curation, writing – review and editing, visualization, supervision, funding acquisition, project administration.

## Conflicts of Interest

The authors declare no conflicts of interest.

## Supporting information




**Supplementary Table**: jfds70812‐sup‐0001‐Table.docx

## Data Availability

The original contributions presented in the study are included in the article, and further inquiries can be directed to the corresponding author.
