# Supplementary material for: Enhancing Gluten‐Free Bread With Whole Flours From Forage Palm, Buckwheat, and Teff: Physicochemical Composition, Technological Properties, and Sensory Evaluation
Source: J Food Sci. 2026 Jan 5;91(1):e70812. doi: 10.1111/1750-3841.70812 (PMC12770811; doi:10.1111/1750-3841.70812)
Supplement: Supplementary file 1 — Supplementary Table: jfds70812‐sup‐0001‐Table.docx [file JFDS-91-0-s001.docx]

**S1.** Formulation of gluten-free bread samples prepared with buckwheat, teff, and forage palm whole flours.

| **Ingredients** | **Percentage (%)** |
| --- | --- |
| Buckwheat, teff and forage palm whole flours | 100 |
| Egg albumin | 5 |
| Sucrose | 4 |
| Instant dry yeast | 2 |
| Palm fat | 4 |
| Iodized kitchen salt | 1.8 |
| Carboxymethylcellulose | 1.5 |
| Phospholipase | 0.003 |
| Transglutaminase | 0.01 |
| α-amylase | 0.001 |
| Xylanase | 0.003 |

All ingredient percentages are expressed on a flour basis, with the flour blends (buckwheat, teff, and forage palm) accounting for 100% of the formulation.
